# Rice JASMONIC ACID OXIDASES control resting jasmonate metabolism to promote growth and repress basal immune responses

**DOI:** 10.1093/plphys/kiaf161

**Published:** 2025-04-24

**Authors:** Simon Ndecky, Ludivine Malherbe, Claire Villette, Véronique Chalvon, Isabelle Meusnier, Dennisse Beltran-Valencia, Nicolas Baumberger, Michael Riemann, Thomas Kroj, Antony Champion, Thierry Heitz

**Affiliations:** Institut de Biologie Moléculaire des Plantes (IBMP) du CNRS, Université de Strasbourg, 67000 Strasbourg, France; Institut de Biologie Moléculaire des Plantes (IBMP) du CNRS, Université de Strasbourg, 67000 Strasbourg, France; Institut de Biologie Moléculaire des Plantes (IBMP) du CNRS, Université de Strasbourg, 67000 Strasbourg, France; PHIM Plant Health Institute, Université de Montpellier, INRAE, CIRAD, Institut Agro, IRD, 34000 Montpellier, France; PHIM Plant Health Institute, Université de Montpellier, INRAE, CIRAD, Institut Agro, IRD, 34000 Montpellier, France; Institut de Biologie Moléculaire des Plantes (IBMP) du CNRS, Université de Strasbourg, 67000 Strasbourg, France; Institut de Biologie Moléculaire des Plantes (IBMP) du CNRS, Université de Strasbourg, 67000 Strasbourg, France; Joseph Gottlieb Kölreuter Institute for Plant Sciences, Karlsruhe Institute of Technology (KIT), 76131 Karlsruhe, Germany; PHIM Plant Health Institute, Université de Montpellier, INRAE, CIRAD, Institut Agro, IRD, 34000 Montpellier, France; DIADE, Institut de Recherche et de Développement (IRD), Université de Montpellier, 34000 Montpellier, France; Institut de Biologie Moléculaire des Plantes (IBMP) du CNRS, Université de Strasbourg, 67000 Strasbourg, France

## Abstract

Catabolic conversions within the jasmonate pathway have substantial consequences on phytohormone signaling output. In dicots, the jasmonic acid oxidase (JAO) catabolic route leads to jasmonic acid (JA) hydroxylation, which limits its conjugation into bioactive jasmonoyl-isoleucine (JA-Ile). Here, we functionally characterized the JAO pathway in rice (*Oryza sativa*) and demonstrated its key function in promoting growth and attenuating JA responses in vegetative tissues. The rice genome encodes 4 JAO-related homologs, 3 of which generate hydroxy-JA in vitro and rescue the high-defense phenotype of the Arabidopsis *jao2-2* mutant. By generating and analyzing a series of single to quadruple rice *jao* mutants, we showed additive effects of cumulative JAO depletion on JA metabolism, basal defense levels, growth inhibition, fitness, and global metabolic reprogramming. The growth of JAO-deficient lines was substantially repressed at the juvenile stage, while the impact was milder in later vegetative development, during which plants opposed enhanced resistance to virulent and avirulent strains of *Magnaporthe oryzae*, the causal agent of fungal blast disease. Moreover, *jao* mutants exhibited slightly reduced fertility and impaired seed filling. Our findings identify the JAO pathway as an integral component of basal JA/JA-Ile homeostasis and an important determinant of the growth–defense tradeoff in rice. The regulatory function of this pathway is conserved in monocots, opening possibilities for selectively modulating basal JA responses in major cereal crops to optimize agronomic traits.

## Teaser

The jasmonic acid oxidase pathway shapes resting jasmonic acid homeostasis and impacts the growth–defense tradeoff in rice.

## Introduction

Jasmonates (JAs) are fatty acid–derived compounds with regulatory functions that impact the entire plant life cycle, from germination to vegetative growth and reproductive success. JAs have critical functions in the interaction of plants with their biotic environment including beneficial and pathogenic organisms, and largely orchestrate a conserved antagonism between growth and defense (Wasternack and Hause 2013; Campos et al. 2014; Guo et al. 2018a). JAs are best characterized as positive regulators of transcriptional responses to herbivores or to microbial pathogens, but recent efforts have uncovered additional functions in the adaptation to numerous abiotic stresses including drought, salt, extreme temperatures or nutrient stress (Kazan 2015; Ali and Baek 2020; Raza et al. 2021).

Synthesis of jasmonic acid (JA), the name-giving compound and hormone precursor, is initiated in plastids upon release of linolenic acid from membrane lipids (Kimberlin et al. 2022), before its oxygenation to an allene oxide intermediate that can be cyclized into the first jasmonate in the pathway, 12-oxo-phytodienoic acid (OPDA). In the major biosynthetic route, a fraction of OPDA is converted to JA in the peroxisomes that will be transferred in the cytosol (Wasternack and Hause 2013; Heitz 2020). Even though exogenous JA triggers extensive transcriptional reprogramming and physiological responses, this precursor acquires its activity only endogenously through JASMONATE RESISTANT 1 (JAR1)-catalyzed conjugation into jasmonoyl-isoleucine (JA-Ile) (Staswick and Tiryaki 2004), which is one among numerous possible metabolic fates (Miersch et al. 2008; Heitz et al. 2019).

Under low hormone levels, JA-responsive transcription factors (TFs) are kept inactive by a family of JASMONATE ZIM-DOMAIN (JAZ) repressor proteins and associated co-repressors (Pauwels and Goossens 2011). When developmental- or stress-induced JA-Ile formation occurs, the conjugate acts as a ligand that promotes the assembly of receptor complexes whose core components are the F-box protein CORONATINE INSENSITIVE 1 (COI1) and various JAZ protein(s). This leads to the ubiquitination of JAZ by the E3 ubiquitin ligase S-phase kinase-associated protein 1—Cullin 1—F-box protein SCF^COI1^ and the subsequent degradation of JAZ by the proteasome, resulting in the transcriptional de-repression of arrays of target genes (Chini et al. 2007; Thines et al. 2007).

**Figure 1.**
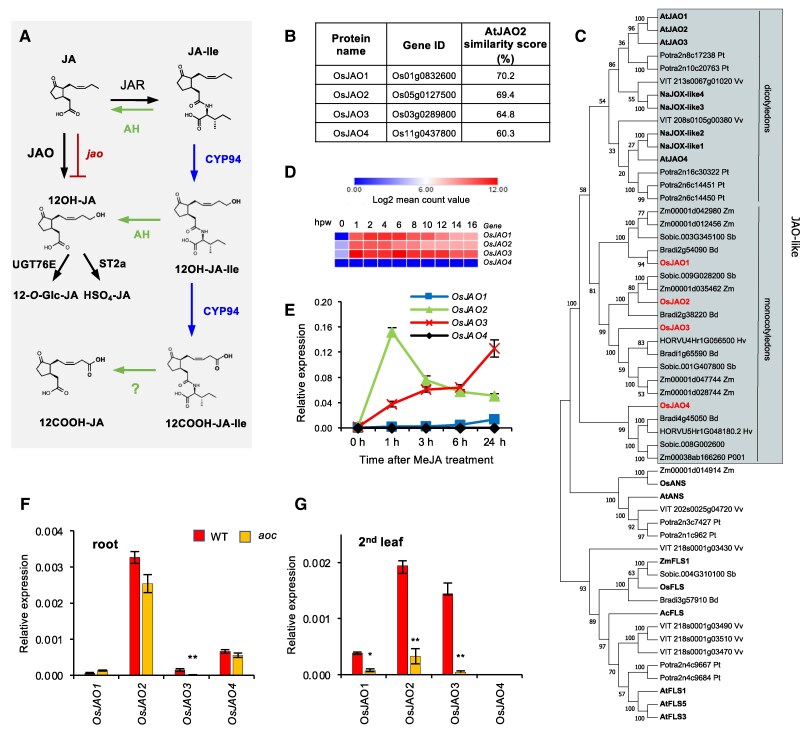
Jasmonic acid/jasmonoyl-isoleucine (JA/JA-Ile) catabolic pathways and identification of candidate *JASMONIC ACID OXIDASE (JAO)* genes in rice. **A)** Simplified metabolic grid of JA/JA-Ile catabolic pathways as initially elucidated in Arabidopsis. Deconjugation steps are labeled in green, AH: amido-hydrolase; conjugate oxidation steps are labeled in blue, CYP94: cytochrome P450 family 94; *jao* mutant labeled in red with an interrupted line; JAR: jasmonate-resistant; ST2A: sulfotransferase 2E; UGT76E: UDP-dependent glycosyltransferase family E. Question mark depicts an uncharacterized step. **B)** Nomenclature, locus ID (RAPdb), and similarity score of the 4 predicted OsJAO proteins to Arabidopsis JAO2 protein. **C)** Phylogenic tree of AtJAO2-related proteins in dicot and monocot plants. Characterized proteins in *A. thaliana* (At) and *N. attenuata* (Na) were displayed along with sequences sharing >35% sequence identity with AtJAO2 from representative species in dicots (*Populus tremula* Potra, *Vitis vinifera* Vv) and monocots (*Brachypodium distachyon* Bd, *Hordeum vulgare* Hv, *O. sativa* Os, *Sorghum bicolor* Sobic, *Zea mays* Zm). Bootstrap values after 100 iterations are shown on nodes. Protein alignment used for constructing the tree is shown in Supplementary Fig. S1. **D)** Kinetic profile of *OsJAO* gene expression in a time series of leaf wounding as obtained by RNAseq. **E)** Kinetic profile of *OsJAO* gene expression in leaves of 10-d-old seedlings after exposure to volatile MeJA. **F** and **G)** Basal expression levels of *OsJAO* genes in **F)** roots or leaves **G)** of wild-type (WT) or JA-deficient *aoc* plants. For **E, F** and **G)**, expression was determined by RT-qPCR and was normalized with the signal from housekeeping genes *UBQ5* and *UBQ10*. **D** to **G)** Data display means of 3 biological replicates with SEM. **F** and **G)** Asterisks indicate a significant difference between genotypes as determined by *t*-test (*P < 0.05; **P < 0.01).

In addition to complex protein interactions in signaling processes, insights during the last decade have established that hormone turnover and other metabolic modifications within the JA biochemical pathway significantly contribute to fine-tuning JA-Ile response output. Knowledge initially gained in Arabidopsis has defined a complex metabolic grid (Fig. 1A) where JA-Ile catabolism is central to generate peculiar JA blend signatures in specific organs or physiological situations (Widemann et al. 2016; Heitz et al. 2019). After its formation and perception, JA-Ile is rapidly and simultaneously turned over by oxidative and deconjugation pathways. Oxidation is catalyzed by 3 JA-regulated cytochrome P450 of the CYP94 subfamily that generates the less active 12OH-JA-Ile and the inactive 12COOH-JA-Ile derivatives (Kitaoka et al. 2011; Koo et al. 2011; Heitz et al. 2012; Jimenez-Aleman et al. 2019; Poudel et al. 2019). JA-Ile as well as its first catabolite 12OH-JA-Ile are also substrates of the amido-hydrolases (AH) IAR3 and ILL6 to generate free JA and 12OH-JA respectively (Widemann et al. 2013; Zhang et al. 2016). The genetic disruption of these metabolic pathways strongly modifies JA-Ile homeostasis with increased steady-state levels and enhanced half-life while their ectopic overexpression depletes the hormone and attenuates downstream responses (Koo et al. 2011; Heitz et al. 2012; Widemann et al. 2013; Zhang et al. 2016; Marquis et al. 2020). Unexpectedly, in Arabidopsis, impaired JA-Ile catabolism does not systematically result in enhanced defense signaling, as such a “super-defense” scenario seems to occur only in some pathway- and stress type-specific combinations, in which repressor feedback mechanisms are not themselves overstimulated (Marquis et al. 2020). In contrast, a more linear output was described in the wild species *Nicotiana attenuata* where silencing of a set of CYP94 enzymes efficiently boosted defense and plant resistance against a generalist herbivore (Luo et al. 2016).

More upstream in the biochemical pathway, diverse JA modifications are known as hydroxylation, sulfation, glucosylation, decarboxylation, or conjugation (Wasternack and Hause 2013); however, their mode of formation or physiological relevance beyond storage was unknown until recently. For example, formation of sulfated or glycosylated JA derivatives is common in plants (Miersch et al. 2008) and requires prior hydroxylation (Fig. 1A). In addition to deconjugation of 12OH-JA-Ile by the AH pathway (Widemann et al. 2013), a direct JA hydroxylation pathway was deciphered in Arabidopsis in the form of 4 JASMONIC ACID OXIDASES (JAO/JOX, Fig. 1, A and C) that belong to a JA-regulated subclade of 2-oxoglutarate-dependent oxygenases. Unexpectedly, the specific suppression of the AtJAO2 isoform is sufficient to redirect metabolic flux toward JA-Ile formation and enhance constitutive JA-Ile-directed responses and antifungal resistance (Smirnova et al. 2017). Multiple *jao* mutations further strengthen JA signaling as evidenced by increased insect attack tolerance and drought survival phenotypes (Caarls et al. 2017; Marquis et al. 2022). Moreover, sulfation of 12OH-JA into HSO_4_-JA by the sulfotransferase ST2a also modifies the defense output, in particular under varying light and shade conditions (Fernandez-Milmanda et al. 2020). These findings highlight the peculiar and potent regulatory function of the JAO pathway and its downstream derivatives, predominantly by defining a metabolic sink impacting JA-Ile formation and action. Consequently, varying the extent of JA hydroxylation activity may be a conserved means plants use to modulate JA-Ile signaling, and this feature deserves to be explored in species of agronomic interest.

Rice (*Oryza sativa*) is a crop species of uppermost importance as the major staple food crop for more than half of the world's population and has been developed as a model in cereal research with ever-increasing resources and technologies. Given its wide genetic diversity and cultivation in diverse habitats, rice is exposed to a tremendous range of biotic and abiotic stresses threatening its productivity. In addition, it suffers from numerous diseases that can cause high-yield losses. Blast disease caused by the hemibiotrophic fungal pathogen *Magnaporthe oryzae* reduces for instance global rice yield by 5% (Savary et al. 2019; Baudin et al. 2024). Its control strongly relies on the use of disease-resistance genes that code for intracellular immune receptors belonging to the class of nucleotide-binding and leucine–rich repeat domain proteins (NLRs), which specifically detect fungal effector proteins (Yin et al. 2021).

In this context, the rice JA hormonal pathway has been extensively investigated, revealing many conserved and some specific features (Dhakarey et al. 2016; Nguyen et al. 2019). For example, some prominent functions of JA in rice are in spikelet development and anther dehiscence (Cai et al. 2014; Xiao et al. 2014), antifungal (Mei et al. 2006; Riemann et al. 2013) or anti-herbivore (Xu et al. 2021; Zeng et al. 2021) defense. In rice, JA-Ile is perceived by functionally diversified COI receptors that have specialized in regulating partially distinct responses. Notably, the divergent COI2 protein harbors important functions in mediating root growth regulation, male fertility, senescence, and antimicrobial defense (Inagaki et al. 2023; Nguyen et al. 2023; Wang et al. 2023). Putative orthologues of most JA biosynthetic and signaling genes have been identified in rice and were functionally characterized in some cases (Nguyen et al. 2019). In contrast, knowledge on jasmonate catabolism and its physiological role is scarce in this species. Rice homologs of CYP94 and AH enzymes and their enzymatic products have been characterized in response to leaf wounding and salt stress (Hazman et al. 2019). Among a series of stress tolerance genes, the most striking gain in salt tolerance was by overexpressing a JA-Ile deactivating enzyme (Kurotani et al. 2015), consistent with the finding that JA signaling is globally detrimental to salt tolerance (Ndecky et al. 2023). Moreover, natural variation in CYP94-catalyzed JA-Ile catabolism was reported in rice and haplotypes with a weaker expression of CYP94C2 where characteristic of accessions better adapted to a temperate climate (Mao et al. 2019). These data provide a link between catabolic plasticity in the JA pathway and peculiar phenotypic characteristics.

To further explore the metabolic regulation of JA signaling and its potential to modulate agronomic traits, we set out to investigate the JAO pathway in rice. Out of 4 *AtJAO2*-related rice genes, 3 were found to be JA-regulated and to encode JAO activity. By generating and analyzing a collection of single to quadruple rice os*jao* mutant lines, we show that JAO activity depletion gradually shifts jasmonate metabolism toward enhanced JA-Ile formation and catabolism, with a likely predominant contribution of OsJAO2/3. Phenotypically, os*jao* mutants display repressed juvenile growth, elevated basal expression of JA marker genes, substantial metabolic reprogramming, and increased resistance to the blast fungal disease caused by *Magnaporthe oryzae*. Our study uncovers the need to tightly control basal JA homeostasis to repress costly JA responses and optimize growth in a cereal species.

## Results

### Rice expresses four *AtJAO2*-related genes

By using the AtJAO2 protein as a query, 4 related sequences were retrieved from the predicted proteome of the rice reference accession Nipponbare (IRGSP-1.0 annotation). According to decreasing similarity with AtJAO2, they were tentatively named OsJAO1 (Os01g0832600), OsJAO2 (Os05g0127500), OsJAO3 (Os03g0289800), and OsJAO4 (Os11g0437800) (Fig. 1B). When constructing a phylogenic tree of sequences originating from representative species from angiosperm lineages, OsJAO sequences formed a monophyletic clade with other monocot sequences in the DOX46 subclade of 2-oxoglutarate/Fe (II)-dependent dioxygenases (2-ODDs) defined by Kawai et al. (2014), adjacent to a group of dicot proteins including JAOs characterized functionally from Arabidopsis (Caarls et al. 2017; Smirnova et al. 2017) and *Nicotiana attenuata* (Tang et al. 2020) (Fig. 1C; Supplementary Fig. S1). The JAO-related subclade of ODDs clustered close to another group formed of flavonoid biosynthetic genes from both monocots and dicot species.

As characterized dicot *JAO* genes are notoriously co-regulated with JA pathway genes (Caarls et al. 2017; Smirnova et al. 2017), we tested the response of the *OsJAO* genes in leaves to either mechanical wounding or MeJA treatment. *OsJAO4* transcripts were not detected in leaves in any tested condition, whereas *OsJAO1*, *2,* and *3* expressions were rapidly induced after wounding, reaching a plateau by 1 h throughout at least 16 h (Fig. 1D). In response to MeJA exposure, contrasted profiles were recorded: *OsJAO2* transcripts peaked early by 1 h before declining, *OsJAO3* increased steadily until 24 h and *OsJAO1* showed a weak and late increase (Fig. 1E). Because basal expression level rather than inducibility was demonstrated to determine biological function in Arabidopsis (Smirnova et al. 2017), we examined precisely the relative transcript levels of *OsJAO* in unstressed rice tissues in WT and JA-deficient *aoc* mutant (Nguyen et al. 2020). *OsJAO2* expressed the highest in roots, followed by *OsJAO4*, whereas *OsJAO3* and *OsJAO1* only showed marginal signal (Fig. 1F). Of note, all genes except *OsJAO3* were equally expressed in both genotypes. In leaf material, *OsJAO2* and *OsJAO3* dominated *OsJAO1* expression, and all 3 genes lost most basal expression in *aoc* (Fig. 1G). These expression data were consistent with a possible role of *OsJAO* genes in JA metabolism.

### Rice *OsJAOs* encode active jasmonic acid oxidases

**Figure 2.**
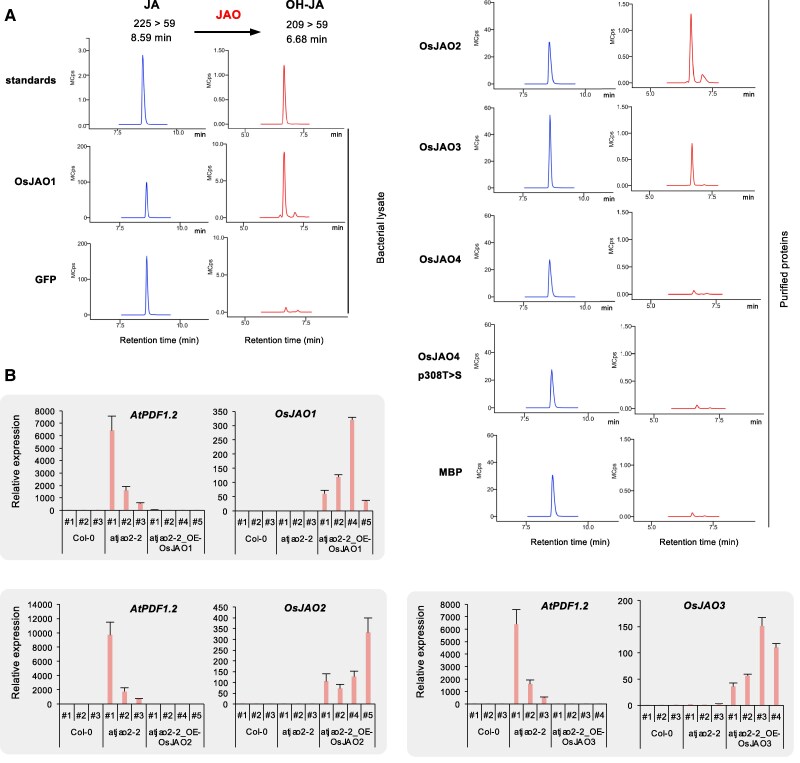
Functional analysis of *OsJAO* genes and proteins. **A)** Catalytic activity of recombinant OsJAO proteins on JA. LC-MS/MS chromatograms of residual JA or produced OH-JA are shown in incubation reactions of JA with bacterial lysate (expressing OsJAO1 or GFP) or affinity-purified proteins (MBP, OsJAO2, OsJAO3 or OsJAO4). Incubations were performed in the presence of the co-substrate 2-oxoglutarate. Control reactions in the absence of the co-substrate are shown in Supplementary Fig. S3. The oxidation product matched the retention time and the mass transition of the authentic 12OH-JA standard. Mcps: megacounts. **B)**  *In planta* complementation assay in Arabidopsis *jao2-2* mutant background expressing *OsJAO* (OE). Three control (Col0 WT and *jao2-2*) and 4 independent T1 transformed plants were analyzed at 3 wks for the impact of *OsJAO* transgene expression on *PDF1.2* marker transcript levels in leaves as determined by RT-qPCR. Expression was normalized with the signal from *EXP* and *TIP41* housekeeping genes. Histograms show mean ± SD of 3 measurements of each plant.

Alignment of the predicted OsJAO protein sequences with those of JA oxidases from Arabidopsis and *N. attenuata* revealed the conservation of almost all residues essential for the binding of the substrates JA and 2-oxoglutarate in structure/function analysis of AtJOX2/JAO2 (Zhang et al. 2021). The only exception was that at position 308 in OsJAO4, the conserved serine (Ser) was substituted by threonine (Thr) (Supplementary Fig. S2). These features suggest that *OsJAO* genes encode active JA oxidases. To test this hypothesis, recombinant OsJAO proteins were expressed in bacteria, and clarified lysates or purified proteins were assayed for jasmonic acid oxidase activity with 2-oxoglutarate (2OG) as a co-substrate. Reaction products were analyzed by liquid chromatography coupled to mass spectrometry (LC-MS/MS). All OsJAO proteins, except OsJAO4, are produced in the presence of JA and 2OG, a compound presenting the characteristics (retention time and mass spectrum) of a synthetic 12OH-JA standard (Fig. 2A). The controls, no-JAO protein or absence of 2OG co-substrate, yielded no oxidized product (Supplementary Fig. S3). An OsJAO4 variant, where the Thr at position 308 was substituted by Ser (OsJAO4p308T>S), also exhibited no JAO activity in vitro, indicating that lack of activity of OsJAO4 is not, or not only due to this substitution (Fig. 2A).

Arabidopsis lines impaired in *AtJAO2* expression display upregulation of JA-dependent defense gene expression in leaves due to a redirection of metabolic flux toward JA-Ile formation and signaling (Smirnova et al. 2017). Taking advantage of this molecular phenotype, we expressed each *OsJAO* gene in the *Atjao2-2* background and assayed *AtPDF1.2* defense marker expression as an in vivo readout of JAO function. In Arabidopsis lines expressing OsJAO1, 2, or 3, *AtPDF1.2* expression was reverted back to low, WT levels (Fig. 2B), while for OsJAO4-expressing lines, no consistent data were obtained. These results indicate that OsJAO1, 2, and 3 modify JA homeostasis and signaling in the Arabidopsis *jao2-2* mutant, suggesting functional conservation between the Arabidopsis and rice proteins.

### Generation of a series of single *jao2* and multiple *jao* rice mutants

To investigate the in vivo functions of OsJAO enzymes, we generated loss-of-function rice mutant lines using the CRISPR–Cas9 gene editing technology. Based on expression data that initially identified *OsJAO1* and *OsJAO2* as the most expressed genes in leaves, we targeted them for generating single mutants using 2 sgRNAs for each gene (Supplementary Fig. S4A). In addition, we prepared a multiplex construct containing one specific gRNA for each *OsJAO* gene interspersed with tRNA^Gly^ sequences (Xie et al. 2015). Supplementary Figure S4B shows the positions of each sgRNA in the respective gene structures. For an unresolved reason, all (T-DNA positive) plants regenerated after transformation with the *OsJAO1* construct exhibited a WT *OsJAO1* sequence, and consequently, no single *Osjao1* ko lines were obtained. Homozygous T1 plants were recovered with one (*jao2* #27) or 2 (*jao2* #55) nucleotide deletions, or one nucleotide insertion in the *JAO2* coding sequence (Supplementary Fig. S5A). From the multiplex construct, homozygous lines with characterized mutations in multiple *JAO* genes were generated (Supplementary Fig. S5A) and were propagated to T3, Cas9-free populations. These various mutations introduce premature stop codons and the synthesis of truncated, inactive proteins lacking essential residues for binding of Fe^2+^ or the 2OG co-substrate (Supplementary Fig. S5B). On the one hand, 2 quadruple mutant lines with frameshift mutations in all 4 genes, named *jao1.2.3.4 #249* and *jao1.2.3.4 #170*, were obtained (Supplementary Fig. S5A). On the other hand, in addition to these genetically well-defined single *jao2* and quadruple knock-out mutants, we also obtained lines with peculiar genetic lesions in *OsJAO3* and/or *OsJAO4* genes, that may preserve the functionality of encoded protein and give preliminary access to unique intermediate mutant genotypes. These alleles whose functional impact is not fully predictable are marked with a * sign after the gene name. For example, a deletion of 3 nucleotides that changes 2 non-conserved amino acid residues was recorded for *OsJAO3:p.61PD>H* in lines jao1.2.3*.4* #67 and jao1.2.3*.4 #137 (Supplementary Fig. S5, A and B). Ectopic expression of this mutated rice protein in the Arabidopsis *jao2-2* mutant reverted high expression of the JA-regulated defense marker *AtPDF1.2* to low, wild-type levels, suggesting OsJAO3:p.61PD > H has retained JAO activity (Supplementary Fig. S6). Similarly, we assume that an in-frame deletion of 3 nucleotides in *OsJAO4* may not alter OsJAO4 activity, if any, in lines jao1.2.3*.4* #67, jao1.2.3.4* #11, and jao1.2.3.4* #13 (Supplementary Fig. S5, A and B). Although incomplete and prone to future enlargements, this series of original rice JAO-defective mutants was explored to reveal the potential functions of OsJAO genes upon macroscopic or molecular phenotyping. For the 4 latter introduced lines with * label, interpretations will be delivered with particular caution.

### Rice *jao* mutants exhibit altered basal jasmonate homeostasis

**Figure 3.**
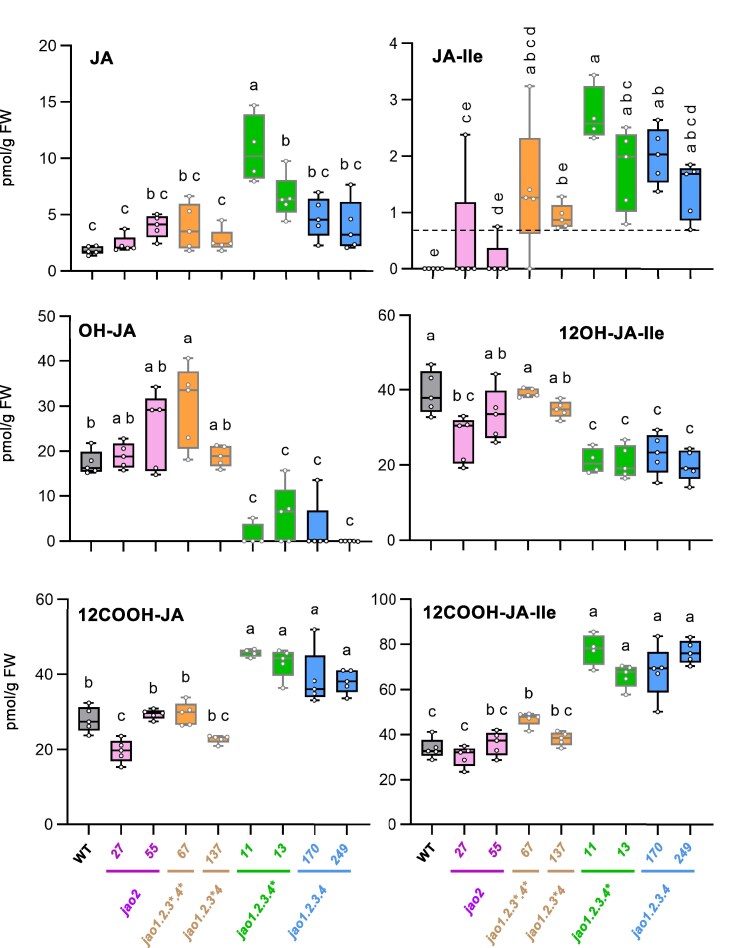
Impact of *jao* mutations on JAs profiles. JAs profiles were analyzed in shoots of WT and a series of *jao* mutants collected from unstressed 9-d-old T3 rice seedlings. Plants were grown on phytoagar in Magenta boxes. JAs were extracted and analyzed using LC-MS/MS. Boxplots show all values as open circles (*n* = 5 biological replicates, except *n* = 4 for jao1.2.3 #11 line) with second quartile, median, and third quartile. JA-Ile limit of quantification (LOQ) was determined at 0.7 pmol/g fresh weight (FW) and is indicated by a dashed line. Data were set to 0 when the signal was below LOQ. LOQ for other JAs are given in Methods. Statistical significance was assessed by one-way ANOVA followed by Tukey post-hoc test with a 95% confidence interval. Different letters above boxplots indicate genotypes that are significantly different (*P* < 0.05). To avoid overloading of graphs, genotype names are reported only on lower panels and apply to all panels.

To evaluate the impact of *jao* mutations on basal JA homeostasis, we quantified 6 JAs in shoots of 9-d-old seedlings of mutant plants grown in the absence of any applied stress. In WT Kitaake plants, basal JA content was around 2 pmol g FW^−1^, JA-Ile was below the limit of quantification (LOQ = 0.7 pmol/g FW), but oxidized derivatives OH-JA, 12OH-JA-Ile, 12COOH-JA-Ile and 12COOH-JA were readily detected in the range of 15–40 pmol g FW^−1^ (Fig. 3). Steady-state levels of JA, the JAO enzyme substrate, were slightly -although not significantly- increased in single and quadruple mutants. The highest impact on JA was in lines simultaneously impaired in JAO1, 2, and 3 functions (#11 and #13). Conversely, an abundance of OH-JA, the direct JAO oxidation product, was weakly or not affected in single *jao2* and jao1.2.3* lines, but was strongly reduced in jao1.2.3.4* #11 and #13 and quadruple mutant lines. The active hormone JA-Ile was detected in all mutants, being most abundant in higher-order mutant lines, and these latter accumulated less of its direct catabolite 12OH-JA-Ile, which is a highly turned-over intermediate in the pathway (Heitz et al. 2012). The carboxylated derivatives, free or Ile-conjugated, which are the most downstream and stable JA catabolites known, distinctly over-accumulated in jao1.2.3.4* and quadruple mutants. These quantitative alterations in mutant lines are consistent with the position of JAO in the JA metabolic grid (Fig. 1A) and the role of OsJAO enzymes in maintaining basal JA homeostasis. They show that cumulative genetic JAO suppression depletes OH-JA formation and promotes JA-Ile accumulation and catabolism in young rice leaves.

### Rice *jao* mutants show developmental phenotypes reminiscent of elevated jasmonate signaling

**Figure 4.**
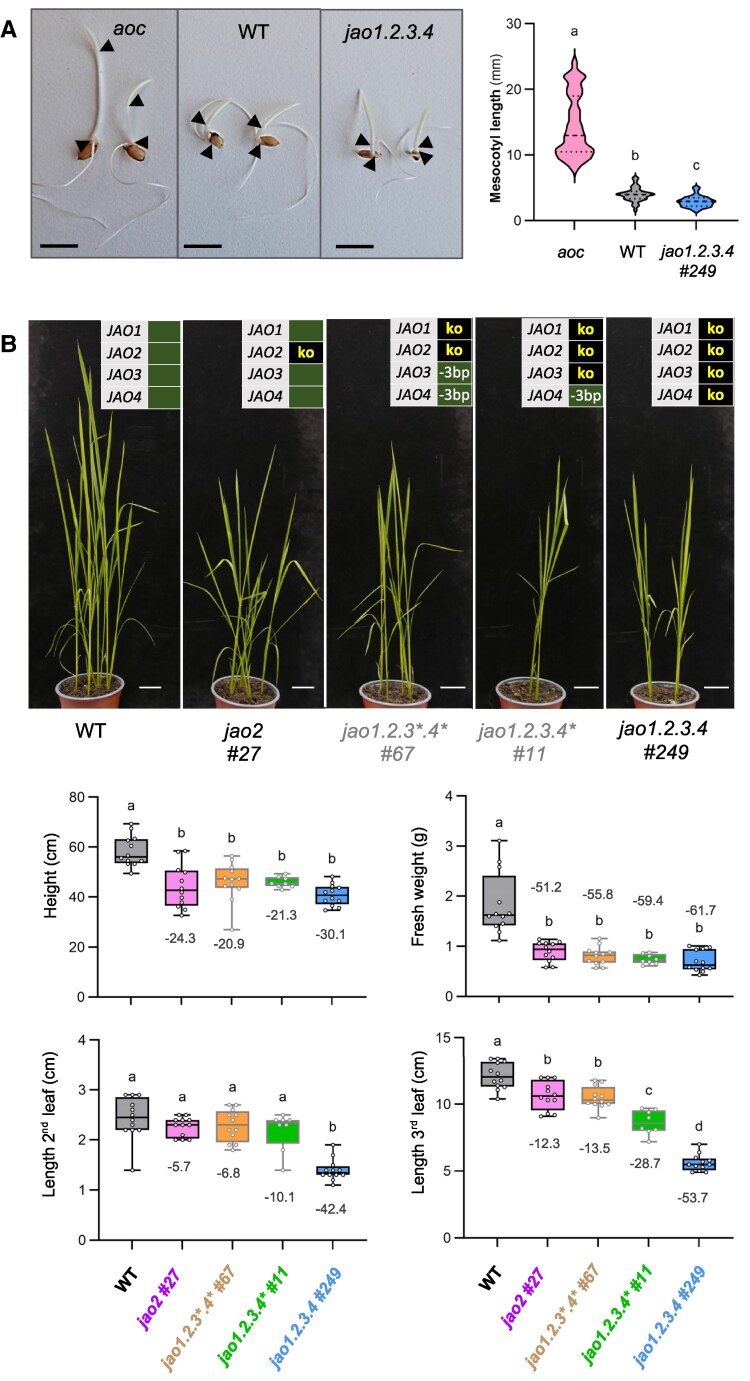
Analysis of growth parameters of rice *jao* mutant lines. **A)** Elongation of mesocotyl under dark conditions in WT, JA-deficient (*aoc*) and *jao1.2.3.4 #137* lines. Seeds were germinated in darkness for 4 days in Petri dishes, and 2 representative seedlings are shown in the left panel. *aoc/aoc* plants were phenotypically identified from an *AOC/aoc* segregating population by their exaggerated mesocotyl elongation. Black arrowheads indicate the limits of the mesocotyl. Scale bar = 1 cm. Right panel represents the measurement of the mesocotyl length of 35–41 seedlings for each genotype. Violin plots represent the distribution of values with the central dotted line representing the median, bottom and top dotted lines indicating the first and third quartile respectively. Statistical significance relative was assessed by Kruskal–Wallis test followed by Dunn's post-hoc test (*P* < 0.05). **B)** Shoot growth phenotypes of T3 plants of single *jao2*, double *jao1.2*, triple *jao1.2.3,* and quadruple *jao1.2.3.4* rice mutants at 4 wks after sowing (*n* = 10–12 per genotype). Scale bar: 2 cm. Indicated genotypes were assessed quantitatively for shoot fresh weight, shoot height, second and third leaf length and displayed in lower panels. Box plots show all values as open circles (*n* = 8–12) with second quartile, median, and third quartile. Numbers indicate the % mean reduction for each genotype relative to WT. Statistical significance was assessed by one-way ANOVA followed by Tukey post-hoc test with a 95% confidence interval. Different letters above boxplots indicate genotypes that are significantly different (*P* < 0.05).

Jasmonate signaling is well-known to affect early seedling development in rice (Riemann and Takano 2008; Svyatyna et al. 2014). For example, under dark conditions, JA-deficient rice (*aoc*) develops an exaggerated long mesocotyl relative to WT, illustrating the repressive action of JA signaling on WT mesocotyl elongation (Ndecky et al. 2023). In this assay, the *jao1.2.3.4 #249* mutant line displayed significantly shorter mesocotyls than WT (Fig. 4A), suggesting that total loss-of-JAO activity may result in increased JA signaling and extreme repression of mesocotyl development in the dark.

Because enhanced basal JA signaling resulted in growth retardation in Arabidopsis *jao* multiple mutants (Caarls et al. 2017; Marquis et al. 2022), we next analyzed rice *jao* mutants for growth characteristics. We first surveyed T3 populations of one-month-old seedlings from single and quadruple mutants. A substantial reduction (up to 30.1%) in plant height and shoot fresh weight (up to 61.7%) was evidenced for *jao2 #27* and *jao1.2.3.4 #249* lines (Fig. 4B). Length of leaf 2 and particularly 3 were strongly affected (−42.4 and −53.7% respectively in quadruple line). For this organ, the multiple mutants jao1.2.3*.4* #67 and jao1.2.3.4* #11 displayed intermediate lengths, potentially reflecting a dosage-dependent impact (Fig. 4B). These observations indicate that severing JAO deficiency may activate JA signaling in elongating tissues leading to increasing repression of vegetative growth. We next examined 2 independent alleles of single *jao2* and *jao1.2.3.4* mutants at a later developmental stage, after 3-month cultivation. Here, the vegetative growth differences were comparable between single *jao2* and quadruple mutant genotypes (Supplementary Fig. S7). Interestingly, the length of a penultimate leaf was reduced maximally by 15.6%, length of internode by 20.7%, suggesting a milder impact of modified JA metabolism on later vegetative development than on young seedlings. At the reproductive stage, we recorded in the 4 analyzed mutant lines a shortening of panicles (from ∼ −6% in single *jao2* to ∼14–15% in quadruple ko lines), a decrease in fertility that was, however, only significant in one quadruple *jao* mutant line, and a significant reduction of seed mass by ∼ 20% (Supplementary Fig. S7).

### JAO deficiency results in elevated basal defense gene expression in rice

**Figure 5.**
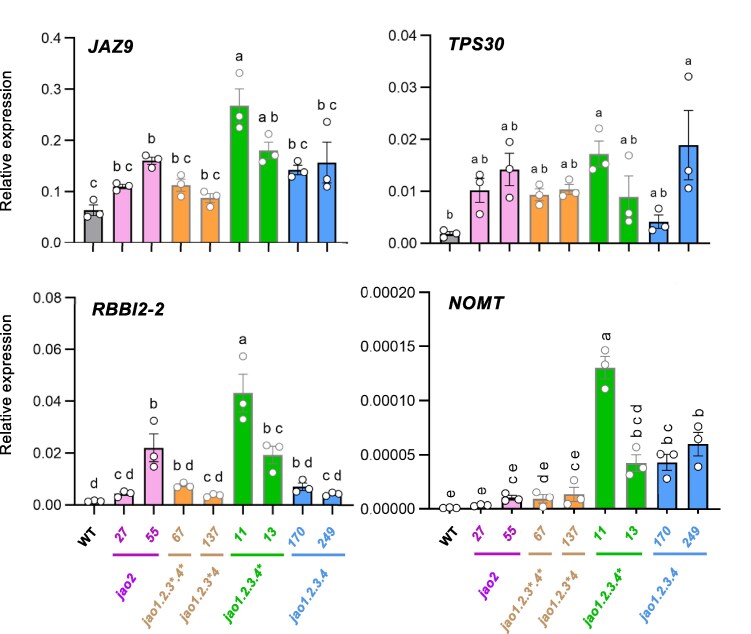
Impact of *jao* mutations on transcript levels of JA-responsive genes in leaves comparatively to WT. The same biological material as in the experiment in Fig. 3 was used for RNA extraction and RT-qPCR analysis. Relative expression was normalized with signals from *UBQ5* and *UBQ10* housekeeping genes. Histograms represent the means of 3 biological replicates with SEM and open circles show individual values. Statistical significance was assessed by one-way ANOVA followed by Tukey post-hoc test with a 95% confidence interval. Different letters above histograms indicate genotypes that are significantly different (*P* < 0.05).

To investigate if enhanced metabolism through JA-Ile impacts hormone signaling as was reported in Arabidopsis and wild tobacco (Caarls et al. 2017; Smirnova et al. 2017; Tang et al. 2020; Marquis et al. 2022), expression of established JA-response markers was quantified in unstimulated 7-d-old rice seedlings of the 8 JAO-deficient lines (Fig. 5) and compared to WT. *JAZ9*, *TPS30*, *RBBII-2,* and *NOMT* genes were significantly upregulated in single *jao2* and quadruple mutant lines. *OsTPS30* is the rice homolog of the maize *ZmTPS10* gene involved in the synthesis of the volatile terpene pheromones (E)-β-farnesene and (E)-α-bergamotene (Schnee et al. 2006). *RBBI-2* is JA-regulated proteinase inhibitor with reported antifungal activity (Qu et al. 2003). NOMT is the methyl-transferase catalyzing the terminal biosynthetic step leading to the flavonoid phytoalexin sakuranetin, which exhibits antifungal and anti-herbivore properties (Liu et al. 2023). Of note, their expression not always reflect the number of mutated *JAO* genes and the change in hormone levels. Maximal transcript levels were achieved generally in jao1.2.3.4* mutants while jao1.2.3*.4* and quadruple *jao1.2.3.4* lines had an intermediate gain in expression. We conclude from this behavior that genetic removal of JA hydroxylation triggers increased defense signaling but that the interaction of *jao* mutations results in complex outputs on target gene expression.

### Rice *jao* mutations gradually impact leaf metabolic profiles

**Figure 6.**
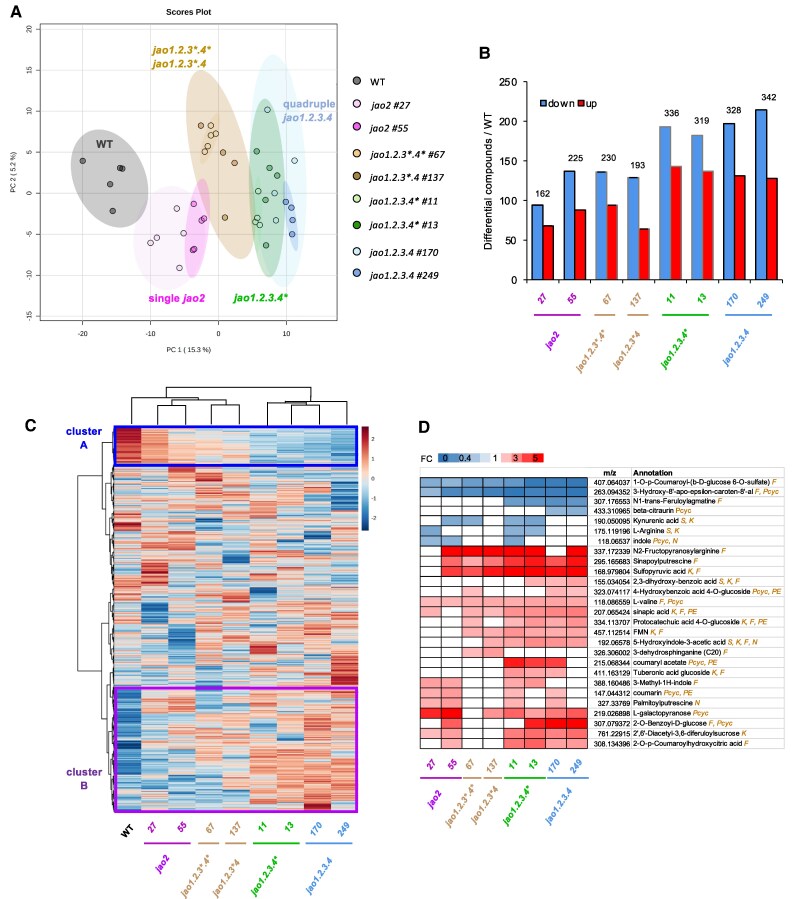
Comparative analysis of non-targeted metabolic profile in WT and eight mutant lines (from single *jao2* to quadruple *jao1.2.3.4*) with impaired expression of *OsJAO* genes. Genes harboring a genetic lesion that may preserve a functional protein are marked with an asterisk (see Supplementary Fig. S5 for details). Leaf material (same as in Fig. 3) was extracted and methanol-soluble metabolic content was analyzed by high-resolution LC-MS/MS. **A)** Principal Component Analysis (PCA) of the distribution of the metabolomes of WT and eight mutant genotypes using 5 biological replicates per genotype except *jao1.2.3 #11*, *jao1.2.4 #137*, *jao1.2.3.4 #170* and *jao1.2.3.4 #249* which had 4 biological replicates. **B)** Number of differential compounds between WT and each indicated mutant genotype among 1,849 total features detected (Supplementary Table S1; Supplementary Table S2). Data recorded from lines with a * allele are shown with gray-framed bars. The numbers above bars indicate the total number of differential compounds. Statistical analysis was conducted with Metaboanalyst 5.0 (https://www.metaboanalyst.ca) tool (−1<log_2_FC>1; *P*-value < 0.1). **C)** Hierarchical heatmap representing the comparative evolution in the abundance of metabolic features in WT and eight JAO-impaired mutant genotypes. Color code refers to the *z*-score for each compound. **D)** Annotated features ranked by global quantitative evolution relative to WT. Differential compounds with putative annotation were determined by pairwise comparison of each genotype with WT. Are displayed only compounds with similar trend change in two lines of same order mutants. Measured *m*/*z* values were used as ID for each compound. Databases producing annotations: F: FoodDB; K: KNApSAcK; N: NPA; PC: PlantCyc; PE: PhenolExplorer; S: Multiple spectral libraries, see Methods section. S symbol elevates annotation confidence to level 2a as defined by Schymanski et al. (2014). FC: Fold Change.

To get a broader picture of the changes in *jao* mutants, we established a global metabolic profile of young leaves of eight JAO-impaired plant genotypes. The overall distribution of signals in a principal component analysis (PCA) revealed that mutants separate into distinct groups with single *jao2* alleles being closest to WT, and quadruple lines diverging further away (Fig. 6A). Interestingly, intermediate lines (jao1.2.3*.4* #67 and jao1.2.3*.4 #137) sharing ko mutations in *OsJAO1* and *OsJAO2* clustered next to *jao2* single lines, whereas lines bearing ko mutations in *OsJAO1*, *2* and *3* barely separated from quadruple mutants. This global assessment indicates an incremental impact of combined *jao* mutations on the medium-polar metabolite content of rice leaves. It also highlights the possibility that *OsJAO4* impairment does not trigger an additional shift in leaf metabolome when combined with other mutations. We detected 1,849 metabolic features in the sample set (Supplementary Table S1) and their relative abundance was submitted to pairwise comparisons between WT and each individual mutant line as shown in Supplementary Table S2. From this latter analysis, the number of differential features (down or up) in each mutant is listed in Fig. 6B. It appears that in all lines, the number of down-regulated compounds exceeds the number of up-regulated ones and that the total number or differentials increases with the number of *JAO* genes mutated, with a plateau reached when *OsJAO1*, *2* and *3* genes are simultaneously impaired. When the features were organized in a hierarchical heatmap, contrasted patterns were revealed among genetic groups (Fig. 6C). For example, a set of features (cluster A) was highest in WT extracts and their abundance gradually decreased from single *jao2* lines to higher-order mutants. Conversely, cluster B groups features of low relative abundance in WT but whose occurrence steadily increased in single to multiple mutants. These findings indicate that *jao* mutations gradually reconfigure sectors of the metabolome in rice leaves with either enhancing or repressing effects on specific sets of compounds. In an attempt to identify some of the JAO-modulated compounds, we interrogate differential compounds with multiple metabolomic databases and established a list of putatively annotated targets (Fig. 6D). This list spanned compounds from several biochemical pathways including indole derivatives, phenylpropanoids, phenolamides or primary metabolites. Of note, some compounds increasing in *jao* mutants were previously reported to display defensive properties. Among overaccumulated compounds are sinapic acid and sinapoylputrescine, belonging to a wound-induced phenolamide class previously described in rice (Tanabe et al. 2016); palmitoylputrescine, displaying antibiotic properties (Brady and Clardy 2004); benzoic acid and hydroxylated derivatives that are fungistatic and antibacterial (Nehela et al. 2021; Zhang et al. 2022); diacetyl-3,6-diferuloylsucrose, an isomer of smilaside M, a potent fungi toxic compound (Zhou et al. 2019). Finally, 5-hydroxyindole-3-acetic acid is a known catabolite of serotonin in animals (Corcuff et al. 2017) and genetic suppression of serotonin in rice triggers resistance to destructive insect pests (Lu et al. 2018).

### Rice *jao* mutations confer increased basal immunity to *M. oryzae*

**Figure 7.**
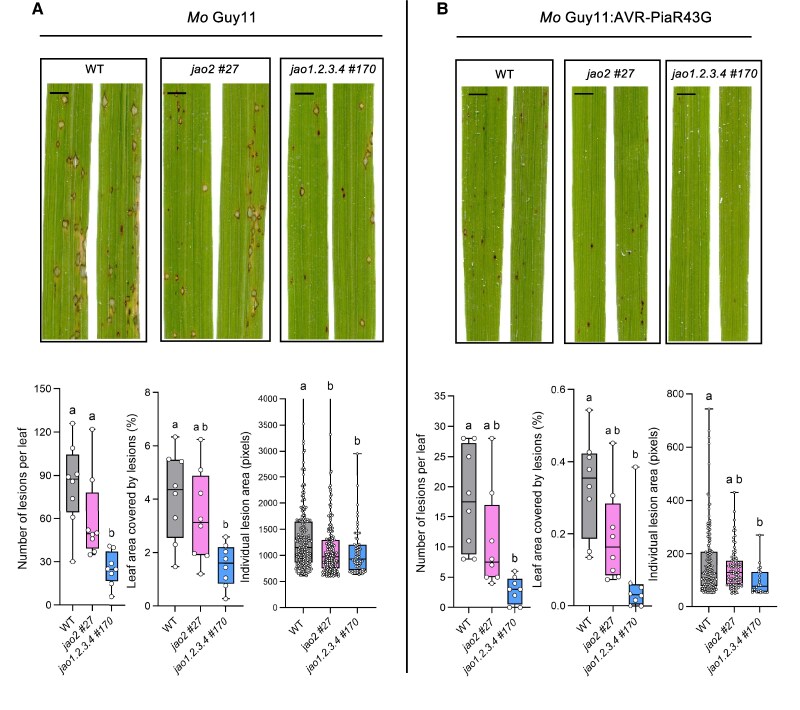
Loss of *OsJAO* genes results in increased basal resistance to *M. oryzae*. Plants of the Kitaake variety (WT and of *jao-2 #27* and *jao1.2.3.4 #170* mutants) were spray-inoculated with spores of the *M. oryzae* strain **A)** Guy11 carrying an empty vector (*Mo* Guy11) or **B)** a Guy11 strain possessing AVR-PiaR43G (Mo Guy11:AVR-PiaR43G). Images were taken by scanning of leaves 7 d after inoculation. Pictures show representative symptoms. Scale bar: 0.5 cm. Plots in lower panels show the number of lesions per leaf (*n* = 8), leaf area covered by lesions, and individual lesion surface (for Guy11 lesions >600 pixels, for Guy11:AVR-PiaR43G lesions >50 pixels, the graph for Guy11 lesions >50 pixels is shown in Supplementary Fig. S8B) as determined by image analysis. The different elements in boxes are center line, median; box limit, upper and lower quartile; wiskers, 1.5× interquartile range; points, outliers. Individual data points are presented as circles. Statistical significance was assessed by one-way ANOVA followed by Tukey post-hoc test with a 95% confidence interval. Different letters above histograms indicate genotypes that are significantly different (*P* < 0.05). Images were digitally extracted for comparison. Equivalent results were obtained in 2 other independent inoculation experiments.

To investigate whether the loss of *OsJAO* genes and its impact on basal defenses influences immunity to a pathogen, we analyzed the interaction of *jao-2* and *jao1.2.3.4* mutant plants with the fungal rice pathogen *Magnaporthe oryzae*. We performed standard whole plant spray inoculation assays (Berruyer et al. 2003) with the *M. oryzae* isolate Guy11 (*Mo* Guy11). As previously reported, *Mo* Guy11 caused typical blast disease lesions on the leaves of Kitaake WT plants (Fig. 7Aand Supplementary Fig. S8A). These lesions were characterized by a dark, brown margin, which is a hallmark of basal immunity, and a gray center, where the fungus sporulates under favorable conditions. In addition to these susceptible lesions, there were also numerous smaller, dark brown lesions, lacking the grey center and that are typical for partial resistance. On the very susceptible variety Maratelli that we used as a positive control, only susceptible type disease lesions were observed (Supplementary Fig. S8A). In the *jao1.2.3.4 #170* mutant, the number of lesions and the leaf area covered by lesions were significantly and strongly reduced as compared to WT plants (Fig. 7A). In the *jao2 #27* mutant, both parameters were also reduced, but to a lesser extent and the differences were not significant. The size of the individual lesions was not altered in both mutants as compared to the WT when all types of lesions were considered together (Supplementary Fig. S8B). However, when large, susceptible lesions were analyzed separately, a significant reduction was observed in both *jao* mutants (Fig. 7A). These results suggest that the loss of the *OsJAO* genes leads to increased basal immunity in rice. A reduced lesion number suggests that *OsJAO* impairment affects the initial invasion of rice epidermal cells by the blast fungus. Reduced size of susceptible lesions indicates that the subsequent development of the fungus inside the leaves is also impaired.

To investigate whether the loss of the *OsJAO* genes influences specific, NLR-mediated resistance, we inoculated the *jao* mutants with an *Mo* Guy11 strain expressing the effector AVR-Pia (Guy11:AVR-Pia). AVR-Pia is recognized in Kitaake by the CNL sensor/helper pair Resistance Gene Analog 4 and 5 (RGA4/RGA5) triggering hypersensitive response (HR) and preventing *Mo* infection (Cesari et al. 2013). The *Mo* Guy11:AVR-Pia strain caused no visible symptoms or very small HR lesions on Kitaake WT and *jao* mutant plants, and no difference in the resistance level was observed between the 3 rice lines (Supplementary Fig. S8C).

To test whether weaker NLR-mediated immune activation would reveal alterations of immunity in the *jao* mutants, we performed inoculation experiments with a *Mo* Guy11 strain expressing an AVR-Pia variant with attenuated RGA5 binding due to the replacement of an arginine residue at position 43 by a glycine (Ortiz et al. 2017). This *Mo* Guy11:AVR-PiaR43G strain triggered weakened resistance on Kitaake characterized by more and larger HR lesions in comparison to the *Mo* Guy11:AVR-Pia wild-type isolate (Fig. 7B, Supplementary Fig. S8D). These extended brown lesions caused by the *Mo* Guy11:AVR-PiaR43G strain on Kitaake leaves were reminiscent of partial resistance. On *jao1.2.3.4 #170* mutant plants, the *Mo* Guy11:AVR-PiaR43G strain caused less lesions and a smaller total lesion area than on WT Kitaake (Fig. 7B). In addition, the area of individual lesions was significantly reduced. On leaves of the *jao2 #27* mutant, the number of lesions, the individual lesion size and the total lesion area per leaf were also reduced, but to a lesser extent than on the quadruple mutant and the differences were again not significant.

We conclude from these experiments that NLR-triggered immunity is not altered in *jao* mutant plants. However, loss of *JAO* gene function resulted in reduced lesion numbers and lesion size in compatible and partially resistant interactions. This finding indicates that the loss of JAO functions limits the frequency of successful fungal penetrations and the subsequent colonization of the leaf by the fungus. These phenotypes could be a consequence of a combination of elevated constitutive defenses in *jao* mutant plants and altered defense activation upon pathogen perception by the basal immune system.

To analyze whether altered basal immunity to Mo is associated with altered antimicrobial defense expression in the *jao2 #27* and *jao1.2.3.4 #170* mutants, we monitored the expression of representative markers of various JA-regulated defense pathways in non-treated plants and plants infected with *Mo* Guy11:AVR-PiaR43G. We could not evidence, in any gene examined, a clear difference in expression amplitude in infected tissue in mutants relative to WT (Supplementary Fig. S8E). In contrast, the predominant genotype differential hallmark was recorded in uninfected plants with higher expression in mutants (in particular in quadruple), as seen for expression of the repressor gene *JAZ8*, the indole biosynthetic gene *IGL* (Frey et al. 2000), or the diterpenoid phytoalexin biosynthetic gene *TPS29* (Sun et al. 2022). This corroborates that *osjao* mutations enhance the defensive capacities of leaves prior to infection.

## Discussion

2-ODDs are involved in virtually every plant hormone metabolic pathway—including abscisic acid, ethylene, gibberellins, salicylic acid, or strigolactones—either in biosynthetic or in catabolic processes. In the JAs pathway, recently characterized JA oxidases have a peculiar position because they define a metabolic diversion that dampens the activation of JA into JA-Ile (Fig. 1A). Although several other JA-modifying enzymes are known (Wasternack and Strnad 2018), *JAO* genetic impairment has revealed its unique function in attenuating basal JA-Ile signaling in Arabidopsis leaves (Smirnova et al. 2017). Transient silencing of homologous genes boosted direct and indirect defenses in the wild tobacco *N. attenuata* (Tang et al. 2020). In both species, impaired JAO function was associated with enhanced tolerance to fungal or insect attacks, as well as better survival to severe drought in Arabidopsis (Caarls et al. 2017; Smirnova et al. 2017; Tang et al. 2020; Marquis et al. 2022). Interestingly, these gains in defensive capabilities were observed without major growth penalty except in the Arabidopsis quadruple *jaoQ* mutant. The JAO pathway therefore bears promising features to modulate the strength of JA-regulated responses, and its potential conservation and impact need to be investigated in crop species.

Here, we addressed the occurrence, activity, and functions of the JAO pathway in rice, a monocot of uppermost agronomic importance. Four predicted homologs of the regulatory AtJAO2 protein were readily identified in the rice genome for functional analysis. Their sequences cluster in a monocotyledon clade along with similar proteins from maize, sorghum, or barley, OsJAO1, 2, and 3 being closest to characterized dicot JAO proteins. OsJAO4 positions further away in a cereal-specific subclade, at the junction with a wide group of proteins acting in flavonoid metabolism. All OsJAO obtained as recombinant proteins but OsJAO4 readily catalyzed oxidation of JA to OH-JA, and the same 3 enzymes reverted the high basal defense phenotype when ectopically expressed in the Arabidopsis *jao2-2* mutant. This demonstrates that the biochemical function of JAO2 is conserved in rice OsJAO1/2/3 proteins and that they attenuate JA-regulated defense responses. The differential responsiveness of *OsJAO1*, *2, and 3* expression to MeJA exposure indicates that each isoform is recruited at distinct timescales, and calls for determining their specific or overlapping sites of expression throughout rice development and adaptation. Despite sharing with the other OsJAOs conserved residues critical for JA substrate binding (Zhang et al. 2021), no consistent functional data could be obtained for OsJAO4. We hypothesize that this protein is either inactive under our conditions or performs a different reaction that may be restricted to roots, as no expression could be detected in leaves.

The use of a multiplex Crispr/Cas9 mutagenic construct allowed to readily obtain an original series of modified plant lines, consisting primarily of 2 alleles of *jao2* mutants and 2 haplotypes of *jao1.2.3.4* quadruple mutants. Despite extensive transformant screening, we could not obtain neat combinations of double and triple mutants; instead, we isolated such mutants harboring additional one-codon in-frame deletions in OsJAO3 and/or OsJAO4, whose impact may be interpreted with more caution because, beyond enzyme activity, these subtle mutations could affect otherwise protein properties., Nevertheless, these latter plant genotypes complemented the set of genetic tools by providing preliminary access to the consequences of stepwise suppression of JAO activity by revealing several phenotypes reminiscent of enhanced JA signaling. The primary observed effect of reduced JAO activity was to redirect metabolic flux toward JA-Ile and its catabolites (Fig. 3). A mild but significant impact was recorded in single *jao2* alleles, that remained stable when *jao1* or *jao4* mutations were added to *jao2* (with jao3*), suggesting a minor contribution of OsJAO1 and OsJAO4 to leaf basal JA homeostasis. In contrast, combined *jao2.3* mutations, either in jao1.2.3.4* #11 or #13, or in 2 quadruple *jao1.2.3.4* lines, resulted in a much stronger metabolic shift, revealing major and redundant contributions of OsJAO2 and OsJAO3 in shaping the basal JAs profile in rice leaves under stress-free conditions, consistent with their predominant basal expression in WT leaves (Fig. 1G). In jao1.2.3.4* and quadruple ko lines, hydroxy-JA, the JAO enzymatic product, is strongly reduced close to the detection limit, and this feature correlates with JA-Ile rising to quantifiable -although modest- basal levels in mutants. This contrasts with the absence of impact reported in Arabidopsis on JA-Ile or OH-JA pools in *jao* mutants (Caarls et al. 2017; Smirnova et al. 2017), indicating differences in JAs homeostasis in the 2 plant species. Also, the intermediate catabolite 12OH-JA-Ile is significantly reduced in rice higher-order mutants, suggesting a minor contribution of CYP94B enzymes in basal JA-Ile oxidative turnover (Hazman et al. 2019), and no compensatory accumulation of OH-JA by its indirect formation pathway (Widemann et al. 2013). In contrast, a common impact of depleting JAO activity in both plant species is the elevated accumulation of downstream catabolites 12COOH-JA-Ile and 12COOH-JA, whose pools are much larger than for JA-Ile itself. This hormonal signature indicates that beyond JA turnover, JAO activity acts as a gatekeeper of JA-Ile formation and catabolism in rice (Fig. 1A; (Smirnova et al. 2017).

The impact of this flux redirection within the JAs metabolic pathway was assessed at the signaling, developmental, and physiopathological levels. First, screening some typical JA gene targets effectively revealed significant upregulation of *JAZ* repressor and basal defense gene expression, a hallmark of higher constitutive JA signaling in leaves. However, their fluctuating expression patterns throughout the mutant series suggest that factors additional to steady-state hormone levels determine the responsiveness of target genes to altered JAO activity. Next, the broader biochemical consequences of reconfigured JA-Ile signaling were addressed by non-targeted metabolomic exploration of the same nine genotypes. PC analysis disclosed remarkable incremental impacts of additive *JAO* impairment on global leaf metabolome (Fig. 6A). The PCA pattern supports the initial assumptions regarding potentially silent mutations within haplotypes and the absence of detected OsJAO4 contribution, *i.e*.: jao1.2.3*4* overlaps with *jao1.2* and jao1.2.3.4* with *jao1.2.3.4* lines. Accordingly, the number of differential compounds relative to WT (Fig. 6B) mirrored the stronger JAs deregulation in higher-order mutants. The hierarchy between genotypes became more apparent from the heatmap which disclosed 2 peculiar clusters of compounds that were progressively reduced (A) or accumulated (B) in mutants (Fig. 6C), indicating a selective impact of JAO activity on the metabolome. Remarkably, when differential abundance of annotated compounds was filtered out relative to WT, typical defense metabolites of the genus *Oryza* did not show up, as was the case for *Brassicaceae-*specific glucosinolate metabolites in the Arabidopsis *jao2* line (Marquis et al. 2022). Instead, elevated contents in phenylpropanoid, phenolamide, indole, or hexose sugar derivatives were recorded in rice, of which several were reported as JA-regulated and/or bearing antimicrobial properties (Fig. 6D). This illustrates that the JAO pathway impacts branches in both primary and specialized metabolism. The overall picture highlights the potential of modulating JAO activity to fine-tune quantitatively JA responses in rice. Despite the absence of single *jao3* and double *jao2.3* mutants in our study, indirect comparative analysis of the available genetic backgrounds showed that most phenotypes are strongest in plant lines impaired in both OsJAO2 and OsJAO3, revealing their overlapping and essential function in regulatory JA oxidation.

Consistent with the preexisting accumulation of JA-regulated defense proteins and metabolites, *jao2* and particularly *jao1.2.3.4* mutants were more resistant to a virulent strain of *M. oryzae*. Specific resistance relying on the detection of the Mo effector AVR-Pia by the paired NLRs RGA4/RGA5 was not altered. However, partial resistance triggered by the detection of the poorly recognized AVR-PiaR43G allele was increased in the *jao* mutant lines. The important role of JA in the basal resistance of rice to *M. oryzae* is well documented (Nguyen et al. 2019), and it has been shown that temperature-dependent variations in JA signaling strongly influence the basal immunity of rice to the blast fungus (Qiu et al. 2022). In particular, the blast fungus deploys virulence functions that interfere with JA signaling in the host plant (Patkar et al. 2015). In our study, we show that increased basal levels of JA-Ile due to impaired catabolism of its precursor leads to a constitutively stronger expression of some immune response genes and provides protection of rice against *M. oryzae*. The induction of immunity responses upon detection of the fungus was not altered but the*in planta* development of the fungus was nevertheless reduced in *jao* mutant plants. This is consistent with the role of *OsJAO* genes in modulating JA levels and signaling mostly in unstressed plants, with possible consequences on the plant holobiont (Carvalhais et al. 2017). The precise impact of JAO deficiency on JAs content in the vicinity of fungal lesions remains unknown, as it is not trivial to produce meaningful quantitative JAs readouts from whole infected leaves: less lesions on highly resistant *jao* mutant leaves (Fig. 7) means less stimulated cells, and may counter-intuitively result in lower JAs abundance than in WT, as was described for *Botrytis*-infected *Arabidopsis* (Smirnova et al. 2017). Also, NLR-mediated specific resistance was not affected in *jao* mutant plants, which is consistent with the mechanism of effector detection and signaling activation by NLRs, which are independent of JA. To what extent JA acts in rice immunity signaling pathways downstream of NLRs is not well established and still needs to be addressed precisely with JA biosynthesis and signaling mutants. Further studies will also be needed to examine the interaction of JAOs with other hormonal pathways (Lahari et al. 2024) or their impact on tolerance to various biotic stresses in rice.

Examination of growth and fitness parameters in seedlings of JAO-impaired lines highlighted interesting features and provided a readout of the importance of intricate JA metabolism on growth/defense tradeoff. While the development of the first leaves was particularly affected by total JAO deficiency (*jao1.2.3.4*), the negative effect on other growth parameters was milder and comparable in the different mutant lines. Interestingly, the vegetative growth penalty was attenuated at later developmental stages, indicating that JAO activity is recruited mostly at early seedling development to optimize growth in rice. In addition, the reduced seed mass of *jao* mutants points to the need to attenuate JA signaling output to maximize seed yield in rice, as reported earlier in other plant species (Baldwin 1998; Guo et al. 2018b). It remains to be determined if this latter outcome results from a reduced supply of photosynthates to the panicle, or if excessive JA signaling impairs flower-specific processes.

In conclusion, we demonstrated that the JAO pathway is biochemically conserved between monocot and dicot species, and most importantly is also endowed with a regulatory function in rice, at the nexus of the growth/defense balance. By diverting metabolic flux upstream of active JA-Ile formation, JAO activity optimizes rice juvenile growth, maximizes seed yield and attenuates the expression of baseline defense programs. While significant redundancy was suspected in leaves, it is possible that individual OsJAO isoforms exert some specific JA repression in discrete organs/tissues or under particular conditions. Their proper identification could provide tools to tailor adequate levels of JA signaling to adapt growth/defense balance and improve specific agronomical traits. Similarly to oxidative JA-Ile catabolism whose natural variation has contributed to rice domestication and propagation to temperate climates (Mao et al. 2019), varying levels of JAO activity may be explored within the wide rice genetic diversity and emerge as a determinant of JA signaling activity.

## Materials and methods

### Rice cultivation

*Oryza sativa* L. ssp. japonica cv. Kitaake was used throughout the study. Seeds were dehusked before being sterilized as described in Ndecky et al. (2023). Seeds were then sown in Magenta boxes containing 0.4% phytoagar (358 mg l^−1^ basal Murashige and Skoog (MS) solution (Duchefa Biochemie) buffered to pH 5.8 with MES. After 7 d in vitro culture in a growth cabinet (Percival Select 41L, Perry, Iowa, USA) under a 12 h/12 h photoperiod with 125 *µ*mol light at 28 °C, the seedlings were transferred to soil and incubated under the same conditions. The growth-related phenotyping was performed after 3 wk of growth on soil in a greenhouse under a 16h-d/8h-night photoperiod, at 28 °C/24 °C with 75% relative humidity. Length of penultimate leaf, internode, and panicle were measured at the mature stage (from 10-wk growth) on the 3 highest tillers from *jao* mutant lines and wild-type plants. The fertility rate was measured on the same tillers as the ratio between the number of fertile spikelets and the total number of spikelets.

### *Magnaporthe oryzae* (*Mo*) infection assays

For inoculation experiments, rice plants were grown in soil in a growth chamber at 28 °C, 16 h light, and 65% relative humidity. *Magnaporthe oryzae* (Mo) strains Guy11, Guy11 transformed with AVR-Pia or AVR-Pia_R43G (Ortiz et al. 2017) were grown as described (Faivre-Rampant et al. 2008). *Mo* conidial spores were collected from 8- to 10-d-old cultures in water with 0.5% gelatin, suspensions of conidia were adjusted to a concentration of 5 × 10^4^ spores ml^−1^ and sprayed onto 3-wk-old rice plants (Berruyer et al. 2003). Inoculated plants were transferred for 16 h in a dark growth chamber regulated at 26 °C and 100% humidity and then placed in normal growth conditions. Symptoms were recorded on the youngest fully expanded leaf of each seedling 7 d after inoculation by imaging and measuring lesions and corresponding leaf areas using the in-house developed program “Leaf Tool” (https://github.com/sravel/LeAFtool).

### Arabidopsis cultivation

All *Arabidopsis thaliana* genotypes used in this study were in a Col-0 ecotype. *atjao2-2* (GK_870C04) T-DNA line was obtained from the Nottingham Arabidopsis Stock Center (NASC, https://arabidopsis.info). Individual ORFs of *OsJAOs* genes were inserted into the pEAQΔP19 plasmid. Recombinant plasmids were mobilized in *Agrobacterium tumefaciens* GV3101 strain before transforming the *jao2-2* line with the floral dip method (Clough and Bent 1998). Primary transformants (T1) were selected on kanamycin-containing MS plates. Kanamycin-resistant seedlings were transferred to the soil under a 16 h/8 h d/night photoperiod (21 °C/16 °C) in a growth chamber. After 2wk of cultivation, unstimulated leaves were collected from 15–20 individual T1 transformants for RNA extraction and defense complementation assay by RT-qPCR. Two independent single copy, complementing lines were conducted to homozygotes in T3 generation.

### Assembly of plasmid constructs

#### Plasmids for JAOs heterologous expression in Arabidopsis

The gene constructs used to overexpress rice OsJAO proteins in the *atjao2-2* Arabidopsis mutant were assembled with the Golden Gate (GG) method into the plasmid pEAQΔP19-GG, a derivative of the pEAQ-HT plasmid lacking the P19 gene (Incarbone et al. 2021). The T-DNA of the pEAQΔP19-GG plasmid has 2 *Sap*I restriction sites located downstream of a 35S promoter (p35S). The coding sequence (ORF) of each individual OsJAO (wild type or mutated) ORF was inserted downstream to the Green Fluorescent Protein (eGFP) ORF. To this end, each *OsJAO* ORF was amplified by PCR from cDNAs produced by retrotranscription of rice plant mRNAs. Forward primer covered about 20 nucleotides upstream of the initiator codon and the reverse primer covered about 20 nucleotides (nt) 3′ of the stop codon. The *Sap*I restriction sites were grafted to the 5′ and 3′ ends of these amplicons through a second PCR. Each OsJAO module was assembled with the eGFP module in the pEAQΔP19-GG plasmid using Golden Gate while ensuring the assembly maintains OsJAO sequences always downstream of eGFP sequence and both OsJAO and eGFP encoded in a unique heterologous CDS. The resulting plasmids were transformed into *E. coli* Top10 strain, selected and then validated by Sanger sequencing.

### Plasmids for bacterial expression

The gene constructs used to express the recombinant proteins 6xHis-MBP-OsJAO4 were assembled by GG into the pETGG plasmid, a Golden Gate derivative from the pET22b(+) plasmid, with 2 *SapI* restriction sites, downstream of the lac operator.

The construct used to express the recombinant 6xHis-MBP-OsJAO2 protein was assembled by Gateway (GW) cloning into the pHMGWA plasmid (Busso et al. 2005) using pDONRzeo as an intermediate. The plasmid constructs used to express the recombinant 6xHis-OsJAO1 and 6xHis-OsJAO3 protein were also assembled into the pHGWA (Busso et al. 2005) plasmid by the GW method.

### Plasmids for rice mutagenesis

#### General strategy

Rice mutagenesis was performed using CRISPR–Cas9 strategy thanks to 2 simplex constructs pUbi-Cas9:sgRNAs-OsJAO1 and pUbi-Cas9:sgRNAs-OsJAO2 used to generate *jao1* and *jao2* single mutants respectively. These 2 plasmids encode mainly the Cas9 enzyme and 2 sgRNAs specific to the targeted gene. A third plasmid pUbi-Cas9:sgRNAs-Multiplex whose T-DNA encodes one sgRNA for each of the 4 *JAO* genes was used to produce different variants of multiple mutants. All 3 recombined plasmids were assembled using the pUbi-Cas9 and pENTR4:gRNA4 vectors based on the methods reported by Zhou et al. (2014) and Xie et al. (2015).

All the primers used in the study are listed in Supplementary Table S3. The CRISPR RNAs (crRNA) crRNA8-OsJAO1 and crRNA9-OsJAO1 were used to target the *OsJAO1* gene. crRNA9-OsJAO1 was first assembled by hybridization of the 24-nt long primers crRNA9-OsJAO1_fw and crRNA9-OsJAO1_rv that are complementary on the 20 nt of their 3′ end that correspond to the sequence of crRNA9-JAO1. The 4 nt in the 5′ of these primers are complementary to the sticky ends generated by the cleavage of pENTR4:gRNA4 by the *Bsa*I enzyme. By insertion between the *Bsa*I sites, crRNA9-JAO1 was placed under the control of the U6p2 promoter and forms, together with the trans-activating crRNA (tracrRNA) downstream of the promoter, sgRNA9-OsJAO1. The pENTR4:sgRNA9-OsJAO1 plasmid formed was subsequently used to insert crRNA8-OsJAO1 between the *Btg*ZI sites. crRNA8-OsJAO1 was assembled in the same way as crRNA9, by hybridization of the complementary primers crRNA8-OsJAO1_fw and crRNA8-OsJAO1_rv. The 4 nucleotides in the 5′ of the latter 2 primers are complementary to the sticky ends generated by the cleavage of pENTR4:sgRNA9-OsJAO1 by the *Btg*ZI enzyme. Thanks to its insertion between the *Btg*ZI sites, crRNA8-OsJOA1 was placed under the control of the U6p1 promoter and forms, together with the tracrRNA downstream of the promoter, sgRNA8. The plasmid pENTR4:sgRNA9-OsJAO1 thus became pENTR4:sgRNAs-OsJAO1. For unknown reasons, recombining pENTR4:sgRNAs-OsJAO1 with pUbi-Cas9 by LR recombination was impossible. As an alternative, we PCR-amplified the assembled construct of the pENTR4:sgRNAs-OsJAO1 plasmid and transferred it into the pENTR1A entry plasmid using BP recombination. LR recombination of the neoformed pENTR1A:sgRNAs-OsJAO1 with the pUbi-Cas9 plasmid generated the vector pUbi-Cas9:sgRNAs-OsJAO1.

crRNA1-OsJAO2 and crRNA4-OsJAO2 were used to target the *OsJAO2* gene. We used the same method as that used to assemble the pUbi-Cas9:sgRNAsOsJAO1 plasmid. In this case, crRNA1-OsJAO2 was placed under the control of the U6p1 promoter and crRNA4-OsJAO2 under the control of the U6p2 promoter.

For the multiplex construct, a single crRNA was used to target each of the 4 *OsJAOs* genes. The *OsJAO1* gene was targeted by crRNA9-OsJAO1, the *OsJAO2* gene by crRNA4-OsJAO2, the *OsJAO3* and *OsJAO4* genes were targeted by crRNA4-OsJAO3 and crRNA1-OsJAO4, respectively (Supplementary Table S3). To facilitate the assembly of the sgRNAs, we reused the pENTR4:sgRNA9-OsJAO1 plasmid in which sgRNA9-OsJAO1 is already assembled in front of the U6p2 promoter. The sgRNAs of the *OsJAO2*, *OsJAO3*, and *OsJAO4* genes were assembled in this plasmid in front of the U6p1 promoter as a polycistronic sequence according to a multiplexing method reported by Xie et al. (2015). Because the assembled plasmid pENTR4:sgRNAs-Multiplex was unable to recombine with pUbi-Cas9 through an LR reaction, the generated sgRNAs construct was PCR-amplified was transferred into the pENTR1a entry plasmid. LR recombination of the neoformed pENTR1a-sgRNAs-Multiplex with pUbi-Cas9 produced the pUbi-Cas9-sgRNAs-Multiplex plasmid used for the production of multiple mutants.

### Plant transformation

#### Production of Arabidopsis transgenic lines

Recombinant plasmids were mobilized into *Agrobacterium tumefaciens* GV3101 strain before transforming the *jao2-2* line with the floral dip method (Clough and Bent 1998). Primary transformants (T1) were selected on kanamycin-containing MS plates. Kanamycin-resistant seedlings were transferred to the soil under a 16 h/8 h photoperiod (21 °C/16 °C) in a growth chamber. After 2-wk cultivation, unstimulated leaves were collected from 15–20 individual T1 transformants for RNA extraction and defense complementation assay by RT-qPCR as described by Smirnova et al. (2017). Two independent single-copy, complementing lines were conducted to homozygotes in T3 generation for each *OsJAO* gene.

#### Production of rice knock-out mutants

Genetic transformation of rice (*O. sativa*, cv. Kitaake) was performed on embryogenic calli based on the protocol described by (Hiei and Komari 2008). Briefly, *A. tumefaciens* bacteria (strain EHA105) transformed with the plasmid constructs pUbi-Cas9:sgRNAs-OsJAO1 or pUbi-Cas9:sgRNAs-Multiplex were co-cultured with rice calli to induce T-DNA transfer into the genome of callus cells. The transformed callus cells were then selected based on their resistance to hygromycin conferred by the *HPTII* gene present on the T-DNA and regenerated into seedlings to constitute the T0 generation of transgenic plants. Genomic DNA was isolated from leaves of T0 plants to confirm the presence of a T-DNA by PCR. Mutations in target genes were then searched for using 2 analytical methods. First, the plants of interest were subjected to High-Resolution Melting (HRM) analysis on a LightCycler 480 II instrument (Roche Applied Science, Penzberg, Germany) to identify individuals with mutations in the target genes. The analysis was performed in a 10-*µ*L reaction with Precision Melt Supermix kit (Bio-Rad) according to the manufacturer's instructions. Second, when positive, the exact nature of the induced mutations in *OsJAO* genes was revealed by Sanger sequencing. When the mutation was heterozygous, an analysis of the chromatograms generated by sequencing with the ICE-Synthego tool (Conant et al. 2022) was necessary to distinguish the different alleles present at the locus concerned. Next, T1 and T2 progeny of these mutation-bearing plant lines were first screened for absence of T-DNA (Cas9-free) and second identified as knock-out (ko) for the targeted *OsJAOs* genes by PCR and resequencing of mutated loci.

### Expression and purification of recombinant proteins

*Escherichia coli* bacteria of the Rosetta 2 pLyS strain transformed with pHGWA:OsJAO1, pHMGWA:OsJAO2, pHGWA:OsJAO3, and pETGG:6xHis-HMP-OsJAO4 were first cultivated at 37 °C and 250 rpm in LB medium to reach the optical density (A_600_) of 0.45 and 0.5. Expression of the recombinant proteins was induced by adding IPTG 0.5 mm and the cultures were further incubated at 20 °C for 4 h. Cells were then collected by centrifugation (20 min, 3,000g, 4 °C), and the bacterial pellets were frozen at −80 °C until processing. Recombinant protein expression was analyzed in total bacteria by western blot using a monoclonal mouse anti-6x-his antibody (Covalab, Bron, France) and goat anti-mouse secondary antibody coupled to horseradish peroxidase (Invitrogen). The signal was recorded using luminescence on a Fusion Fx instrument (Vilber Lourmat, Marne-la-Vallée, France). For JAO activity assays to be performed on purified proteins, the thawed bacterial pellets were resuspended in lysis buffer (300 mm NaCl, 20 mm imidazole in 50 mm Tris–HCl pH 7.5) at 20 A_600_ units mL^−1^. Bacteria were lysed by sonication (VibraCell 75115, Bioblock Scientific), and cell debris was removed by centrifugation (15 min, 17,000g, 4 °C). The clarified lysate was filtered at 0.22 *µ*m, and the proteins of interest were purified by affinity chromatography by affinity chromatography on an Äkta pure Fast Protein Liquid Chromatography system equipped with a Histrap FF crude 1 ml IMAC (immobilized metal affinity chromatography) column (Cytiva). Equilibration and binding and washes were done with 50 mm Tris pH 8, 300 mm NaCl, 5% glycerol, 25 mm imidazole, and elution with 50 mm Tris pH 8, 300 mm NaCl, 5% glycerol, and 500 mm imidazole. Eluted fractions were analyzed by Coomassie Blue–stained polyacrylamide gel or by Western blot. The protein concentration of eluates was estimated by quantification with the Bradford reagent using a bovine serum albumin (BSA) calibration range. For the enzymatic tests performed directly with the bacterial lysates, the collected cell pellets were resuspended in buffer devoid of imidazole (300 mm NaCl, 5 mm dithiothreitol (DTT) in 50 mm Tris–HCl pH 7.5), and cells lysed by sonication (VibraCell 75115, Bioblock Scientific). Cell debris was then removed by centrifugation (15 min, 17,000g, 4 °C), and clarified lysates were used for enzymatic assay.

### *In vitro* JA oxidation assay

#### Assays with purified enzymes

Each assay was performed with 10 *µ*g purified OsJAO protein in 200-*µ*L reactions consisting of 100 *µ*M JA substrate, 50 mm Tris–HCl pH 7.5, 5 mm DTT, 13.3 mm 2-oxoglutarate, 13.3 mm ascorbate, 0.67 mm FeSO_4_ and 200 *µ*g BSA. The reactions were incubated at 30 °C for 1 h before being stopped with 20% (v/v) HCl 1 m. The reaction products were subsequently extracted with one volume of ethyl acetate. The organic upper phase (200 *µ*L) was transferred in a new tube and dried under nitrogen flow before redissolving in 150 *µ*L methanol. The extracts were analyzed using ultra-high-performance liquid chromatography coupled to tandem mass spectrometry (UPLC-MS/MS) for the detection of JA and its oxidized form OH-JA produced by JAOs as described by Smirnova et al. (2017).

#### Assays with clarified bacterial lysates

For OsJAO1 that did not bind properly to affinity resin, assays were conducted with 150 *µ*L bacterial lysate in 300 *µ*L reactions including 30 *µ*M JA substrate, 50 mm Tris–HCl of pH 7.5, 5 mm DTT, 50 mm 2-OG, 50 mm ascorbate, 0.67 mm FeSO_4_, and 15 *µ*g BSA. The mixtures were incubated at 30 °C for 1 h, and the reaction products were extracted and analyzed following the same procedure used after the enzymatic assays with the purified proteins.

### Extraction of metabolites

Between 35 and 40 mg of frozen fine powder of the leaf plant material were extracted with 80% methanol containing 0.5% acetic acid and 1 *µ*M 9,10-dihydro-JA-Ile, and 100 nm Prostaglandin A1 (Cayman Chemicals, Ann Arbor, Michigan, USA). The material was extracted in 2-ml screw-capped microtubes in the presence of 12 *μ*L mg^−1^ extraction solution by homogenizing with an orbital grinder (Precellys Evolution, Bertin Technologies, France) in the presence of glass beads (2 cycles of 30 s at 6,500 rpm separated by 30 s pause). After 1 h of incubation at 4 °C on a rotating wheel, the cell debris was sedimented by 2 successive centrifugations (10 min, 11,000g, 4 °C), and 120 *µ*L of the supernatant was transferred to LC vials containing a glass insert (N9, Machery-Nagel, Düren, Germany).

### Jasmonate profiling

Jasmonate profiling was performed by LC-MS/MS as described by Marquis et al. (2022) on extracts described earlier. Limits of quantification (LOQ) were determined on extracts of jasmonate-deficient rice *aoc* leaves (Nguyen et al. 2020) spiked with known amounts of reference compounds. LOQ were (in pmol/g FW): JA, 1; JA-Ile, 0.7; OH-JA, 20; 12OH-JA-Ile, 1; 12COOH-JA-Ile, 1; 12COOH-JA, 4.

### Metabolome analysis

Untargeted metabolite analysis was performed on the same extracts that used for JA profiling on an UltiMate 3000 UHPLC system (Thermo Fischer Scientifc, Illkirch, France) coupled to the ImpactII high-resolution Quadrupole Time-of-Flight (QTOF) spectrometer (Bruker, Wissembourg, France). Chromatographic separation was performed on an Acquity UPLC HSS T3 column (2.1 × 100 mm, 1.8 *µ*m, Waters) coupled to an Acquity UPLC HSS T3 pre-column (2.1 × 5 mm, 1.8 *µ*m, Waters). The raw data extracted from these analyses were processed with MetaboScape 4.0 software (Bruker): the molecular characteristics were considered and grouped into “buckets” containing one or more adducts and isotopes of the detected ions with their retention time and MS/MS information when available. The parameters used for the definition of the buckets are: a minimum intensity threshold of 5 000, a minimum peak length of 3 spectra, a signal-to-noise ratio (S/N) of 3, and a correlation coefficient threshold of 0.8. The ion [M + H]^+^ was allowed as a primary ion, and the ions [M + Na]^+^, [M + NH4]^+^, and [M + K]^+^ were allowed as possible seed ions. Biological replicates of the same genotype were grouped together and only the buckets found in 80% of the samples in a group were extracted from the raw data. The resulting list was annotated using SmartFormula to generate a raw formula based on the exact mass of the primary ions and the isotopic pattern. The maximum allowable variation in mass (Δ*m*/*z*) was set at 3 ppm, and the maximum mSigma value (assessing isotopic model conformity) was set at 30. To name the resulting formulae, analyte lists were derived from FooDB (http://foodb.ca), KNApSacK (http://www.knapsackfamily.com/KNApSAcK/), PlantCyc (https://plantcyc.org/), PhenolExplorer (http://phenol-explorer.eu/), NPA, and spectral libraries (Bruker MetaboBASE, Mass Bank, LipidBlast, MSDIAL LipidsDB). The parameters used for the annotation with the analyte lists are the same as for the SmartFormula annotation. The resulting annotations are at level 3 (analyte lists) and level 2 (spectral libraries) of the Schymanski classification (Schymanski et al. 2014). Statistical analysis was performed with MetaboAnalyst 5.0 (www.metaboanalyst.ca) with 5 samples per group using peak areas as the reference unit. Data were normalized by sum before log transformation and Pareto scaling. Compounds were considered statistically differential between the 2 groups using thresholds of *P*-value ≤0.1 and fold change ≥2 or ≤−2.

### RNA extraction and gene expression profiling

Leaf samples were ground using the glass-bead homogenizer before RNA isolation using TRizol Reagent according to manufacturer instructions. For RT-qPCR analysis, cDNA was synthesized with Superscript IV Reverse Transcriptase (Thermofisher) using 2 *µ*g of RNA. qPCR was performed using 20 ng of cDNA on a LightCycler 480 II instrument (Roche Applied Science, Penzberg, Germany) as described by Berr et al. (2010). The expression levels of the different rice target genes were normalized against the expression level of the reference genes *UBQ5* (Os01g0328400) and *UBQ10* (Os02g0161900) and the expression level of the Arabidopsis target gene *AtPDF1.2* was normalized against the expression level of the reference genes *AtEXP* (At4g26410) and *AtTIP41* (At4g34270). The sequences of all primers used are listed in Supplementary Table S3.

### RNAseq analysis

WT Kitaake seeds were grown for 7 d post-germination in the growth chamber at 26 °C under 12/12 h, under 70% humidity. The first developed leaf was wounded 3 times with forceps. The leaves were then sampled following a kinetic including 10 time points, at 0, 1, 2, 4, 6, 8, 10, 12, 14, and 16 h post-wounding. For each time point, 5 leaves were pooled and 3 biological replicates were analyzed. Total RNA was extracted from rice leaf samples using the RNeasy Plant mini kit (Qiagen) with the addition of an on-column DNase I digestion. cDNA libraries preparation and sequencing were performed using the Illumina NovaSeq platform generating 150-bp paired-end reads (Novogene, Cambridge, UK). Count files were generated as previously described (Cheaib et al. 2024). DEGs were detected using EdgeR running in the DIANE Shiny application (Cassan et al. 2021). DEGs were defined as log_2_Fold Change (log_2_FC) >1 or <−1, False Discovery Rate (FDR) <0.01. The RNAseq dataset was deposited in the NCBI Gene Expression Omnibus repository under the accession GSE279814.

### Genomic DNA extraction

A small piece of frozen leaf from each rice plant was placed in wells of 96-sample plates and dry-ground in the TissueLyser II (Qiagen) using steel balls. Samples were then ground a second time in the presence of 500 *µ*L Edwards buffer (250 mm Tris HCl of pH 7.4, 250 mm NaCl, 25 mm EDTA, and 0.5% SDS), the plate centrifugated (maximum speed, 10 min) to sediment the cell debris. One hundred microlitres of the supernatant from these cell extracts was recovered and mixed with 75 *µ*L of isopropanol to precipitate DNA. After centrifugation (maximum speed, 15 min), the DNA pellet was washed once with 70% ethanol before being resuspended in 50 *µ*L of distilled water.

### Protein sequence analysis

Protein sequences used in the phylogenetic classification of rice JAOs and their sequence study were extracted were retrieved from the comparative genomics portal Phytozome (https://phytozome–next.jgi.doe.gov). The MEGA-X software (Kumar et al. 2018) was then used to mine protein sequences. The multiple sequence alignment was performed with the Muscle method (Edgar 2004), and the phylogenetic tree was drawn with the “Maximum likelihood” method.

### Accession numbers

Sequence data from this article can be found under accession numbers_ OsJAO1 (Os01g0832600), OsJAO2 (Os05g0127500), OsJAO3 (Os03g0289800), and OsJAO4 (Os11g0437800).

## Supplementary Material

kiaf161_Supplementary_Data

## Data Availability

The data underlying this article are available in the article and in its online supplementary material.

## Supplementary data

The following materials are available in the online version of this article.

**Supplementary Figure S1.** Multiple sequence alignment of selected AtJAO2-related protein sequences from representative dicot and monocot species.

**Supplementary Figure S2.** Alignment of predicted OsJAO protein sequences with partial sequences of functionally characterized JAO proteins from Arabidopsis and *Nicotiana attenuata*.

**Supplementary Figure S3.** Negative controls for the incubation reactions of recombinant OsJAO from bacterial lysate (OsJAO1 and GFP) or affinity-purified proteins (OsJAO2, 3 and 4) shown in Fig. 2A.

**Supplementary Figure S4.** CRISPR–Cas9 plasmid constructs used for generating *Osjao* mutant lines and the positions of target sequences for each guide RNA in *OsJAO* genes.

**Supplementary Figure S5.** Representation of mutations (indels) identified in *OsJAO* genes in different rice mutant lines in the T1 generation, and the truncated proteins encoded by these mutated genes.

**Supplementary Figure S6.**  *In planta* assay of OsJAO3:p.61PD>H functionality.

**Supplementary Figure S7.** Analysis of the growth parameters of rice mutant lines impaired in *OsJAO* expression at the T3 generation.

**Supplementary Figure S8.** Phenotypic characterization of the interaction of rice *jao* mutant lines with virulent and avirulent strains of the fungal pathogen *Magnaporthe oryzae*.

**Supplementary Table S1.** Metabolic features detected in the extracts of nine-day-old rice seedlings.

**Supplementary Table S2.** Differentially accumulated compounds (DC) between the eight *JAO*-deficient mutant lines and WT Kitaake.

**Supplementary Table S3.** Primers used in the study.

## Dive Curated Terms

The following phenotypic, genotypic, and functional terms are of significance to the work described in this paper:

IAR3 Gramene: AT1G51760
IAR3 Araport: AT1G51760

ILL6 Gramene: AT1G44350
ILL6 Araport: AT1G44350

protein CHEBI: CHEBI:36080

COI1 Gramene: AT2G39940
COI1 Araport: AT2G39940

JAZ8 Gramene: AT1G30135
JAZ8 Araport: AT1G30135

OsJAO1 Gramene: Os01g0832600
OsJAO1 Araport: Os01g0832600

OsJAO2 Gramene: Os05g0127500
OsJAO2 Araport: Os05g0127500

OsJAO3 Gramene: Os03g0289800
OsJAO3 Araport: Os03g0289800

OsJAO4 Gramene: Os11g0437800
OsJAO4 Araport: Os11g0437800
