# Supplementary material for: Rice JASMONIC ACID OXIDASES control resting jasmonate metabolism to promote growth and repress basal immune responses
Source: Plant Physiol. 2025 Apr 24;198(1):kiaf161. doi: 10.1093/plphys/kiaf161 (PMC12076411; doi:10.1093/plphys/kiaf161)
Supplement: kiaf161_Supplementary_Data [file kiaf161_supplementary_data.zip › Suppl Figures.pdf]

Supplementary Figure S1 :

Multiple sequence alignment of selected AtJAO2-related protein sequences from representative dicot and monocot species. Secondary structure elements are indicated on top (helices with squiggles, ß-strands with arrows and turns with TT letters). Positions that are identical are highlighted by white letters and colored background; positions with >70% similarity are highlighted with a grey background. Letter colors indicate chemical family of residues: red: negatively charged; turquoise: positively charged; pink: hydrophobic; blue: aromatic; brown: non-charged lateral chain; orange: other. Alignment was performed with MUSCLE (Edgar, 2004) and displayed using the ESPript server (Robert and Gouet, 2014). Protein data entry for AtJAO2 is 6LSV. Supports Figure 1C.

Edgar, R.C. (2004). MUSCLE: multiple sequence alignment with high accuracy and high throughput. Nucleic Acids Res **32**: 1792–1797.

Robert, X. and Gouet, P. (2014). Deciphering key features in protein structures with the new ENDscript server. Nucleic Acids Res. **42**: W320–W324.

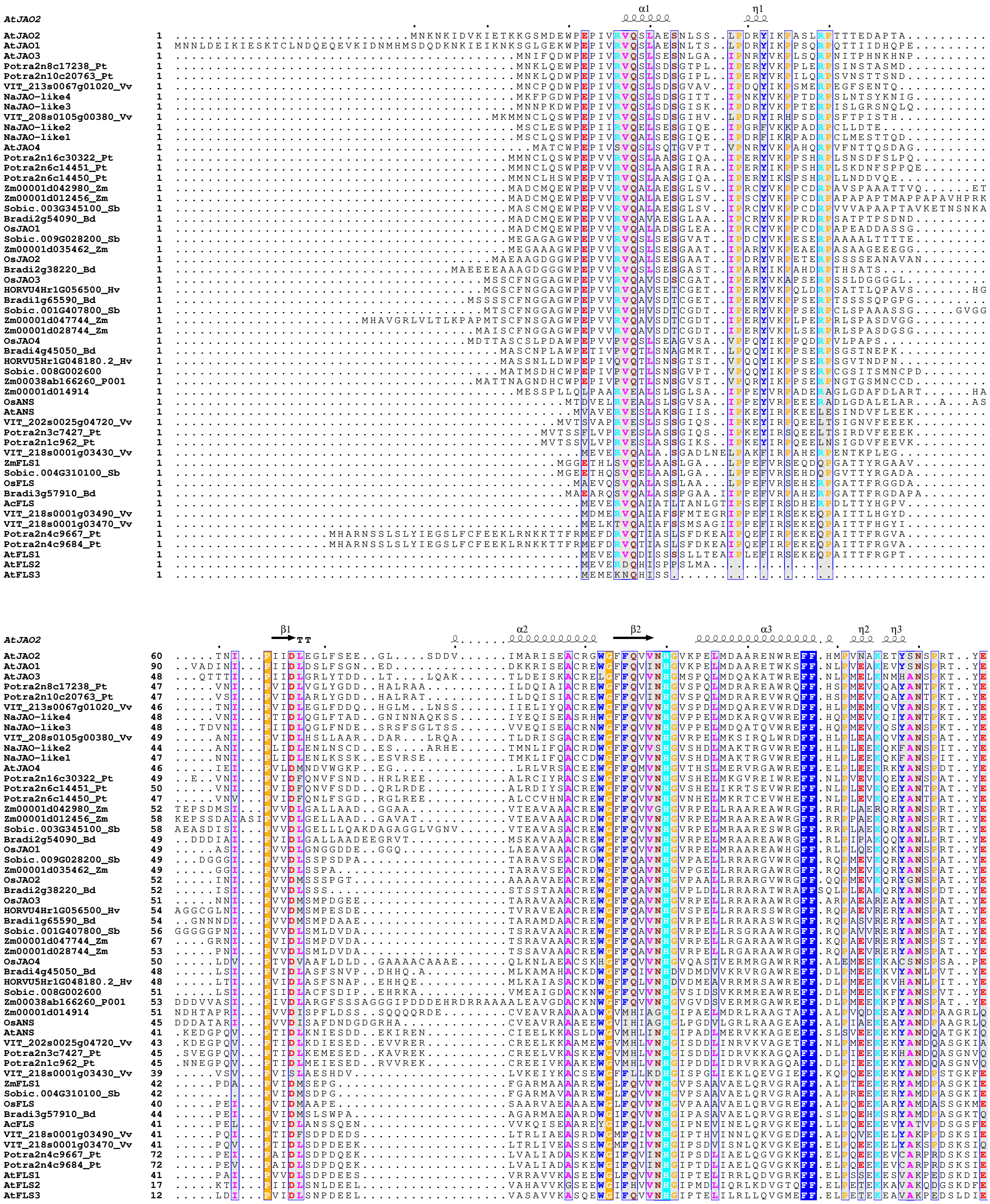
AtJAO2
α1
η1
β1
α2
β2
α3
η2
η3
TT
AtJAO2
AtJAO1
AtJAO3
Potra2n8c17238_Pt
Potra2n10c20763_Pt
VIT_213s0067g01020_Vv
NaJAO-like4
NaJAO-like3
VIT_208s0105g00380_Vv
NaJAO-like2
NaJAO-like1
AtJAO4
Potra2n16c30322_Pt
Potra2n6c14451_Pt
Potra2n6c14450_Pt
Zm00001d042980_Zm
Zm00001d012456_Zm
Sobic.003G345100_Sb
Bradi2g54090_Bd
OsJAO1
Sobic.009G028200_Sb
Zm00001d035462_Zm
OsJAO2
Bradi2g38220_Bd
OsJAO3
HORVU4Hr1G056500_Hv
Bradi1g65590_Bd
Sobic.001G407800_Sb
Zm00001d047744_Zm
Zm00001d028744_Zm
OsJAO4
Bradi4g45050_Bd
HORVU5Hr1G048180.2_Hv
Sobic.008G002600
Zm00038ab166260_P001
Zm00001d014914
OsANS
AtANS
VIT_202s0025g04720_Vv
Potra2n3c7427_Pt
Potra2n1c962_Pt
VIT_218s0001g03430_Vv
ZmFLS1
Sobic.004G310100_Sb
OsFLS
Bradi3g57910_Bd
AcFLS
VIT_218s0001g03490_Vv
VIT_218s0001g03470_Vv
Potra2n4c9667_Pt
Potra2n4c9684_Pt
AtFLS1
AtFLS2
AtFLS3

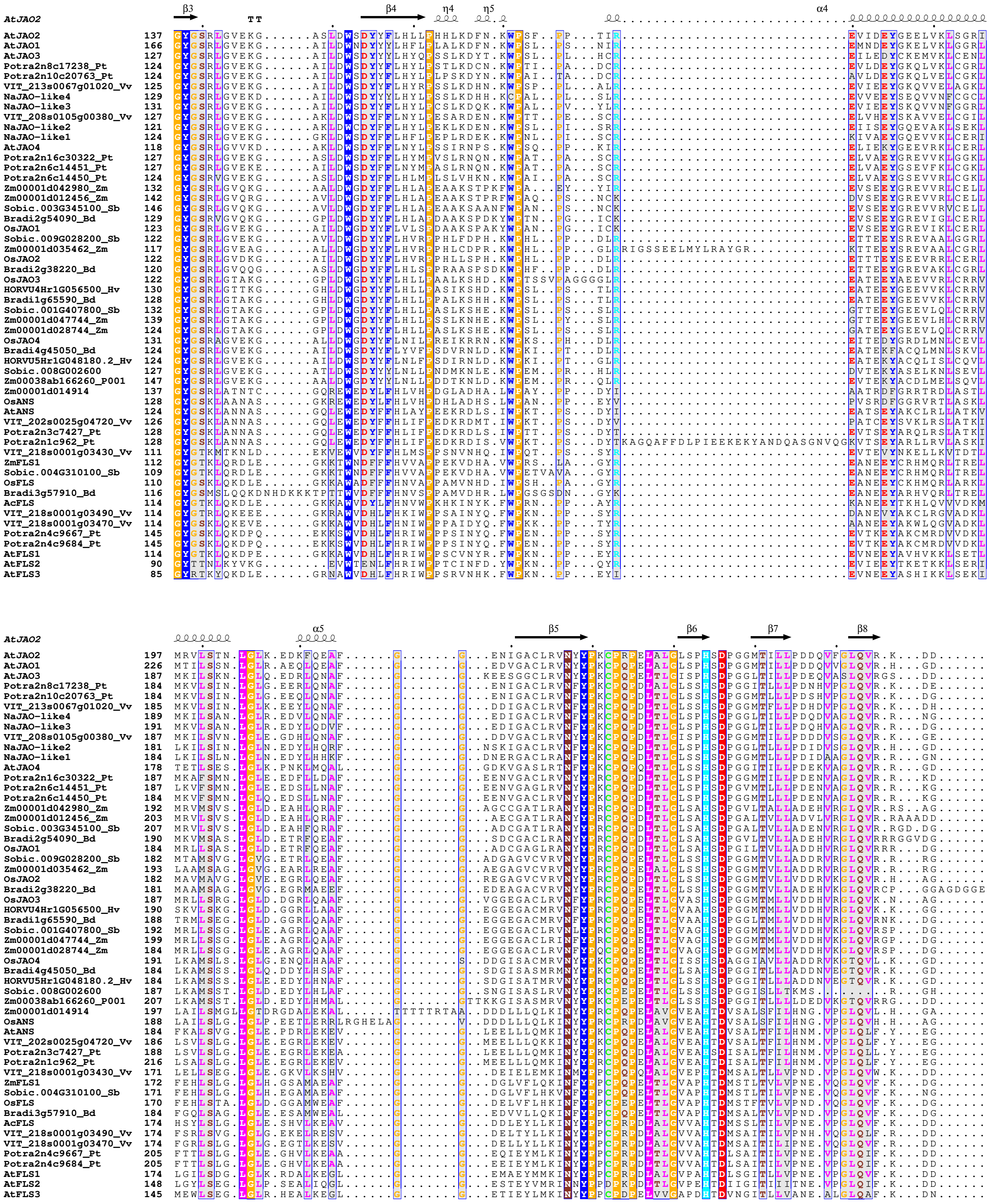
AtJAO2
β3
TT
β4
η4
η5
α4
AtJAO2 137
AtJAO1 166
AtJAO3 127
Potra2n8c17238_Pt 124
Potra2n10c20763_Pt 124
VIT_213s0067g01020_Vv 125
NaJAO-like4 129
NaJAO-like3 131
VIT_208s0105g00380_Vv 127
NaJAO-like2 121
NaJAO-like1 124
AtJAO4 118
Potra2n16c30322_Pt 127
Potra2n6c14451_Pt 127
Potra2n6c14450_Pt 124
Zm00001d042980_Zm 132
Zm00001d012456_Zm 142
Sobic.003G345100_Sb 146
Bradi2g54090_Bd 129
OsJAO1 123
Sobic.009G028200_Sb 122
Zm00001d035462_Zm 117
OsJAO2 122
Bradi2g38220_Bd 120
OsJAO3 122
HORVU4Hr1G056500_Hv 130
Bradi1g65590_Bd 128
Sobic.001G407800_Sb 132
Zm00001d047744_Zm 139
Zm00001d028744_Zm 124
OsJAO4 131
Bradi4g45050_Bd 124
HORVU5Hr1G048180.2_Hv 124
Sobic.008G002600 127
Zm00038ab166260_P001 147
Zm00001d014914 137
OsANS 128
AtANS 124
VIT_202s0025g04720_Vv 126
Potra2n3c7427_Pt 128
Potra2n1c962_Pt 128
VIT_218s0001g03430_Vv 111
ZmFLS1 112
Sobic.004G310100_Sb 109
OsFLS 110
Bradi3g57910_Bd 116
AcFLS 114
VIT_218s0001g03490_Vv 114
VIT_218s0001g03470_Vv 114
Potra2n4c9667_Pt 145
Potra2n4c9684_Pt 145
AtFLS1 114
AtFLS2 90
AtFLS3 85
AtJAO2
α5
β5
β6
β7
β8
AtJAO2 197
AtJAO1 226
AtJAO3 187
Potra2n8c17238_Pt 184
Potra2n10c20763_Pt 184
VIT_213s0067g01020_Vv 185
NaJAO-like4 189
NaJAO-like3 191
VIT_208s0105g00380_Vv 187
NaJAO-like2 181
NaJAO-like1 184
AtJAO4 178
Potra2n16c30322_Pt 187
Potra2n6c14451_Pt 187
Potra2n6c14450_Pt 184
Zm00001d042980_Zm 192
Zm00001d012456_Zm 203
Sobic.003G345100_Sb 207
Bradi2g54090_Bd 190
OsJAO1 184
Sobic.009G028200_Sb 182
Zm00001d035462_Zm 193
OsJAO2 182
Bradi2g38220_Bd 181
OsJAO3 187
HORVU4Hr1G056500_Hv 190
Bradi1g65590_Bd 188
Sobic.001G407800_Sb 192
Zm00001d047744_Zm 199
Zm00001d028744_Zm 184
OsJAO4 191
Bradi4g45050_Bd 184
HORVU5Hr1G048180.2_Hv 184
Sobic.008G002600 187
Zm00038ab166260_P001 207
Zm00001d014914 197
OsANS 188
AtANS 184
VIT_202s0025g04720_Vv 186
Potra2n3c7427_Pt 188
Potra2n1c962_Pt 216
VIT_218s0001g03430_Vv 171
ZmFLS1 172
Sobic.004G310100_Sb 171
OsFLS 170
Bradi3g57910_Bd 184
AcFLS 174
VIT_218s0001g03490_Vv 174
VIT_218s0001g03470_Vv 174
Potra2n4c9667_Pt 205
Potra2n4c9684_Pt 205
AtFLS1 174
AtFLS2 148
AtFLS3 145

```
AtJAO2                       β9  TT  β10  α6  β11  β12  TT  β13  η6  β14  α7  α8

AtJAO2                 269  .TWITVKPHPHAFIVNIGDQIQILSNSTYKSVEHRVIVNSDKERVSLAFFYNPKSDIPI.QPLQELVS.TH.N.PPLY..P.PMTFDQY...RLFIRTQG
AtJAO1                 298  .TWITVNPLRHAFIVNIGDQIQILSNSKYKSVEHRVIVNSEKERVSLAFFYNPKSDIPI.QPMQQLVT.ST.M.PPLY..P.PMTFDQY...RLFIRTQG
AtJAO3                 261  .AWITVEPAPHAFIVNMGDQIQMLSNSIYKSVEHRVIVNPENERLSLAFFYNPKGNVPI.EPLKELVT.VD.S.PALY..S.STTYDRY...RQFIRTQG
Potra2n8c17238_Pt      256  .NWITVKPAPHAFIVNIGDQIQVLSNATYKSVEHRVIVNSSEERVSLAFFYNPKSDVPI.EPLKELVA.PH.R.PPLY..P.AMTFDEY...RLFIRMRG
Potra2n10c20763_Pt     256  .NWITVKPVPHAFIVNIGDQIQVLSNATCKSVEHRVMVNSSEERLSLAFFYNPRSDIPI.EPLKELVA.PD.R.PPLY..P.AMTFDEY...RLFIRMRG
VIT_213s0067g01020_Vv  257  .KWITVKPAPHAFIVNIGDQIQVLSNAIYKSVEHRVMVNSLKERVSLAFFYNPKSDILI.EPVKALVT.PD.T.PALY..P.GMTFDQY...RLYIRMRG
NaJAO-like4            261  .DWITVKPASHAFIVNIGDQIQVLSNGIYKSVEHRVMVNSAEERVSLALFYNPRSDLMI.EPAKEVVT.PE.N.PPLY..P.PMTFDDY...RLYIRTKG
NaJAO-like3            263  .DWITVKPASHAFIVNIGDQIQVLSNAIYKSVEHRVIVNSADERVSLAFFYNPSGDLMI.EPAKELVT.QH.N.PPLY..P.PMTFNEY...RLYMRTKG
VIT_208s0105g00380_Vv  259  .HWVTVEPIPDALIVNLGDQIQVISNAIYKSVEHRVIVNSIKERVSLAYFYNPKGDLLI.EPAKKLVS.KD.R.PASY..S.AMTFDQY...RLFIRQSG
NaJAO-like2            254  .NWLTVKPVPNAFVVNIGDQIQVLSNANYKSVEHRVIVNSAKERVSLAFFYNPGGDILI.KPADELVT.ED.R.PALY..R.PTTFNEY...RALIRTKG
NaJAO-like1            257  .NWLTVKPVPNAFIVNIGDQIQVLSNAMYKSVEHRVIVNSAKERVSLAFFYNPGGDKLI.KPADELVT.ED.R.PALY..F.PTTFNKY...RALIRTKG
AtJAO4                 251  .GWVTIKSVPNALIVNIGDQLQILSNGIYKSVEHQVIVNSGMERVSLAFFYNPRSDIPV.GPIEELVT.AN.R.PALY..K.PIRFDEY...RSLIRQKG
Potra2n16c30322_Pt     260  .SWVTVKPAPNAFIINIGDQIQVLSNAIYQSVEHRVIVNSDKDRVSLAFFYNPKSDLLI.EPSKELVT.VD.R.PALY..P.PMTFDEY...RLYIRTKG
Potra2n6c14451_Pt      260  .SWLTVKPIPNAFIINVGDQLQVLSNGIYQSVEHRVIVNSNRDRVSLALFYNPKNDSII.EPSKEFVS.DD.R.PALY..P.PVRFDEY...RLYVRTKG
Potra2n6c14450_Pt      257  .SWLTVKPIPNAFIINIGDQIQVLSNAIYQSVEHRVIVNSNKDRLSLALFYNPKSDLLL.EPCKELLT.KD.Q.PALY..K.PMTFDEY...RLTIRIKG
Zm00001d042980_Zm      266  .DWVDVRPVRDAFIVNVGDQVQIMSNSVYKSVEHRVVVNAEEERISLAIFYNPKGDVPI.SPAPELVT.AA.KLPPLY..P.TMTFDEY...RLYVRSKG
Zm00001d012456_Zm      278  ..WVTVQPVRDAFIVNVGDQVQILSNSVYKSVEHRVVVNAEEERISLALFYNPKGDVPI.APAPELVA.AA.SLPALY..P.TMTFDEY...RLYVRNKG
Sobic.003G345100_Sb    281  .EWVTVQPVRDAFIVNVGDQVQILSNSVYKSVEHRVVVNAEEERISLALFYNPKGDVPI.SPAPGLVT.AG.NLPALY..P.PMTFDEY...RLYIRNKG
Bradi2g54090_Bd        266  .DWVTVQPVPDAFIVNVGDQIQIMSNSVYKSVEHRVIVNAMEERISLALFYNPRGDLPI.APASELVT.RG.R.PSLY..PRPMTFDEY...RVYIRKNG
OsJAO1                 257  .HWVTVQPLPDAFIVNVGDQIEILSNSMYKSVEHRVIVNAEEERISLALFYNPRGDVPV.APAPELVT.PE.R.PSLY..YRPMTFDEY...RVYVRKNG
Sobic.009G028200_Sb    255  .AWVTVDPVPDAFIVNVGDQIQVLTNATYRSVEHRVMVNAADERLSVALFYNPKSDLPL.APMPELVS.PD.R.PALY..K.PMTFDEY...RMYIRRKG
Zm00001d035462_Zm      266  .AWVTVDPVPDAFIVNVGDQIQVLTNATYRSVEHRVVVNAADERLSVALFYNPRSDLPL.APMPELVS.PD.R.PALY..C.PMTFDDY...RMYIRRKG
OsJAO2                 255  .AWVTVDPVPDAFIINVGDQIQVVTNALYRSVEHRVVVNAAEERLSIATFYNPRSDLPV.APLPELVS.PE.R.PPLY..S.PMTFDDY...RLYIRRNG
Bradi2g38220_Bd        261  DEWVTVDPLPGALIVNVGDQIQVLTNAEYRSVEHRVMANAGAERLSVALFYNPRSDIPL.SPMPELVSLPE.K.PPLY..E.PMTYDQY...RLYIRREG
OsJAO3                 260  .HWITVDPVPDAFIVNIGDQIQVLSNAVYKSVEHRVTVSAAEERLSLAFFYNPRSDLPL.APMPELVAPPA.R.PALY..P.PMTFDEY...REHIRRCG
HORVU4Hr1G056500_Hv    263  .QWITVDPVPDAFIVNVGDQIQVLSNAAYKSVEHRVTVSAAEERLSLAFFYNPRSDVPV.APMAELVS.PE.R.PALY..P.EMTFDEY...RARIRQRG
Bradi1g65590_Bd        262  .HWITVDPVPDAFIVNVGDQIQVLSNAAYKSVEHRVTVSAAEERLSLAFFYNPRSDLPV.APMPEL.....G.PPLY..P.EMTFDEY...RAHIRRRG
Sobic.001G407800_Sb    266  .QWIIVDPVPDAFIVNVGDQIQVLSNASYKSVEHRVTVSAAEDRLSMAFFYNPRSDLPI.APMPELVG.PG.R.PALY..P.EMTFDEY...RVFIRQRG
Zm00001d047744_Zm      273  .QWIIVDPVPDAFIVNIGDQIQVLSNAAYKSVEHRVTVSATEDRLSMAFFYNPRSDLPI.APMAELVG.PG.R.PALY..P.EMTFDEY...RVFIRQRG
Zm00001d028744_Zm      258  .QWIVVEPVPDAFIVNVGDQIQVLSNAAYKSVEHRVTVSAAEDRLSMAFFYNPRSDLPI.APMAELVG.PD.R.PALY..P.EMTFDEY...RVFIRQRG
OsJAO4                 264  .TWYTVQPIPNAFLVNVGDQIQIISNDKYKSVEHRAVASSDDARFTVAFFCNPSGNLPI.GPAAQLVS.SQ.S.PALY..T.PIVFDEY...RRFSRRRG
Bradi4g45050_Bd        257  .SWVTVPPIPASFLVNVGDQLQILSNGRYRSAEHRALANSNKDRFTIAFFCNPQCDLPI.APSSQLVG.PE.S.PALY..QKPVTFDEY...RKYIRTKG
HORVU5Hr1G048180.2_Hv  257  .AWVTVQPVPGSFLVNVGDQIQIMSNGRYRSVEHRALANSDKERFTIAFFCNPRGDLPV.APASELVG.PG.S.PALY..HKAVTFDDY...RKYMRTKG
Sobic.008G002600       252  ........................SNGAYKSVEHRALATSGDDRLTIAFFCNPCGDLPI.APAAQLVG.PE.S.PAVYGQP.AITFNEY...RKYVRTKG
Zm00038ab166260_P001   283  .TWVTVHPIPGAFLVNVGDQIQILSNGAYKSVEHRALASSGHDRLTIAFFCNPCGDLPI.VPAPQLVG.PE.S.PAVYGQP.PITFNEY...RKYVRTKG
Zm00001d014914         278  .RWVTARHEPGTIIVHVGDALEILSNGRYTSVLHRGLVNREAVRISWVVFCEPPPDSVLLHPLPELVT.EG.H.PARF..T.PRTFKQHLDRKLFKKKQQ
OsANS                  266  .SWVTARPEPGTIVVHVGDALEILTNGRYTSVLHRGLVSRDAVRLSWVVFCEPPPESVLLQPVPELLA.DGAD.KPLF..A.PRTFKQH...VQRKLFEK
AtANS                  256  .KWVTAKCVPDSIVMHIGDTLEILSNGKYKSILHRGLVNKEKVRISWAVFCEPPKDKIVLKPLPEMVS.VE.S.PAKF..P.PRTFAQH...IEHKLFGK
VIT_202s0025g04720_Vv  258  .KWVTAKCVPNSIIMHIGDTIEILSNGKYKSILHRGLVNKEKVRISWAVFCEPPKEKIILKPLPETVS.ET.E.PPLF..P.PRTFSQH...IQHKLFRK
Potra2n3c7427_Pt       260  .KWITAKCVPNSIIMHIGDTVEILSNGKYKSIIHRGLVNKEKVRISWAVFCEPPKEKIILKPLAEIVT.EA.E.PPLF..P.PRTFAQH...IEHKLFRK
Potra2n1c962_Pt        288  .KWVTAKCVPNSIIMHIGDTVEILSNGKYKSILHRGVVNKEKVRISWAVFCEPPKEKIILKPLEETVT.EA.E.PPLF..P.PRTFAQH...LAHKLFRK
VIT_218s0001g03430_Vv  242  .NWVVVDYLPNALFVHVGDQIEVLSNGKYKSVLHRSTVDKERTRMSWAVFCAPPHKAMI.GPLPELVD.EP.N.PAKY..S.TKTFAEY...RYRKFNKL
ZmFLS1                 243  .QWYEAKYVPDALIVHIGDQIEIFSNGAYKAVLHRTTVNKEKTRMSWPMFVEPPGELVV.GPHPKLVT.EE.S.PAKY..K.AKKYKDY...QHCKINKL
Sobic.004G310100_Sb    242  .HWYDAKYVPDALIVHIGDQIEIFSNGVYKAVLHRTTVNKEKTRMSWPVFVEPPGELVV.GPHPKLVT.DE.R.PAKY..K.AKKYKDY...QHCKINKL
OsFLS                  241  .HWYDVKYVPDALIIHIGDQIEILSNGRYKAVLHRTTVDKDRTRMSWPVFVEPPPEHVV.GPHPQLVT.DG.S.PAKY..K.AKKFKDY...RHCKINKL
Bradi3g57910_Bd        255  .RWHDVRYVPGALIIHIGDQIEILSNGRYKAVLHRTTVSKDKTRMSWPVFVEPPAEHAV.GPHPQFVT.DE.N.PAKY..K.AKKFRDY...KHCKINKL
AcFLS                  245  .HWFDAKYIPNALICHIGDQLEILSNGKYKSVLHRTTVNKEKSRMSWPVFCSPPGDTMI.GPLPQLVN.DG.N.PPKF..K.TKKYKDY...AYCKINKL
VIT_218s0001g03490_Vv  245  .HWFDVKYIPNALVIHIGDQLEILSNGKYKSVLHRTTVKKDMTRMSWPVFLEPPPELAI.GPLPKLTS.ED.N.PPKY..K.KKRYCDY...VYCKLNKI
VIT_218s0001g03470_Vv  245  .HWFDVKYISNALVIHVGDQLEILSNGKYKAVLHRTTVNKEKTRMSWPVFLEPPSDQVV.GPLPQLVN.EE.N.PARY..K.TKKYSDY...VYCKLNKI
Potra2n4c9667_Pt       276  .QWIEAKYIPDALIIHIGDQIEILSNGKYKAVLHRTTVAKDRARMSWPVFLEPPGELVV.GPLPQFIN.ND.N.PPKF..K.TKKFKDY...TYCKLNKL
Potra2n4c9684_Pt       276  .QWIEAKYIPDALIIHIGDQIEILSNGKYKAVLHRTTVAKDRARMSWPVFLEPPGELVV.GPLPQFIN.ND.N.PPKF..K.TKKFKDY...TYCKLNKL
AtFLS1                 245  .HWFDAEYIPSAVIVHIGDQILRLSNGRYKNVLHRTTVDKEKTRMSWPVFLEPPREKIV.GPLPELTG.DD.N.PPKF..K.PFAFKDY...SYRKLNKL
AtFLS2                 219  .HWLDVHYIPSSITVNIGDQIMVC............................................................................
AtFLS3                 216  .QWIDAEYTTSGIIVIIGDQFLRMSNGKYKSVEHRAKMDKEKTRISWPVFVESSLDQVF.GPLPELITGDE.N.VPKF..K.PYVYKDY...KFRKLKKL


AtJAO2                       α9

AtJAO2                 358  PQGKSHV.ESHISPR................................
AtJAO1                 387  PRGKSHV.ESHISPR................................
AtJAO3                 350  PRSKCHI.DELKSPR................................
Potra2n8c17238_Pt      345  PSGKSQV.ESLKSPR................................
Potra2n10c20763_Pt     345  PCGKSQV.ESLKSPR................................
VIT_213s0067g01020_Vv  346  PRGKSQV.ESLKSPR................................
NaJAO-like4            350  PQGKSQLRQSNKSPT................................
NaJAO-like3            352  PQGKSQLGESQKSSI................................
VIT_208s0105g00380_Vv  348  LWGKSQV.ESLKCSQ................................
NaJAO-like2            343  PSGKSQV.ESLKSPR................................
NaJAO-like1            346  PSGKSQI.ESLKSPR................................
AtJAO4                 340  PCGKNQV.DSLLLTR................................
Potra2n16c30322_Pt     349  PCGKKQV.ESLKSPRPCDER...........................
Potra2n6c14451_Pt      349  LCGKEQV.ESLTIS.................................
Potra2n6c14450_Pt      346  PCGKKQV.ESLKSPGQCN.............................
Zm00001d042980_Zm      356  ARGKAQI.EALKGQASPEN............................
Zm00001d012456_Zm      367  ARGKAQI.EALKGQGTPESQL..........................
Sobic.003G345100_Sb    371  ARGKAQI.EALKGQASPEK............................
Bradi2g54090_Bd        356  PKGKAQL.EGLKAAQAVPAENELN.......................
OsJAO1                 347  PKGKAQL.EALKGQSITQNNE..........................
Sobic.009G028200_Sb    344  PRGKSQV.DSLKATAGGGVNASTS.......................
Zm00001d035462_Zm      355  PRGKSQV.DSLKVADAVDADADADGRRCY..................
OsJAO2                 344  PRGKSQV.DRLAAAAATIPNSTTTTQ.....................
Bradi2g38220_Bd        352  PQGKAQV.DSLKASSSQERP...........................
OsJAO3                 350  LSGKAQL.QSQQMATAICGAPPAATAFSSSSSSASSLAR........
HORVU4Hr1G056500_Hv    352  LSGKAHL.QSLQHACSSVAS...........................
Bradi1g65590_Bd        347  LSGKAQL.QALQTAARSARAAS.........................
Sobic.001G407800_Sb    355  LAGKAQ.........................................
Zm00001d047744_Zm      362  LAGKAQL.ESMQKAAATAADGGRRQLVHQLAATEHIISRASWSVSDA
Zm00001d028744_Zm      347  LAGKAQL.ESLQVKATAGGGGCSAAAASSTS................
OsJAO4                 353  LKGKSQL.EAMKNSKVH..............................
Bradi4g45050_Bd        347  PSGRKQI.LSINSSMQPDPSA..........................
HORVU5Hr1G048180.2_Hv  347  PSGKTQV.QSISSTMQPGPAA..........................
Sobic.008G002600       320  ARGRAQV.ESLSLSLSLPVSSSSTTTTT...................
Zm00038ab166260_P001   374  ARGRAQV.ESLSISTTSTSTTNNNNT.....................
Zm00001d014914         371  HKAKAEEEDGGNGDHHRHEPPPQTN......................
OsANS                  357  LKDQQDN.NAAAASNGMRTK...........................
AtANS                  346  EQEELVSEKND....................................
VIT_202s0025g04720_Vv  348  TQEALLSK.......................................
Potra2n3c7427_Pt       350  TQDSLLPTKAN....................................
Potra2n1c962_Pt        378  TQDSLTPPKAD....................................
VIT_218s0001g03430_Vv  331  PQ.............................................
ZmFLS1                 332  PM.............................................
Sobic.004G310100_Sb    331  PM.............................................
OsFLS                  330  PM.............................................
Bradi3g57910_Bd        344  PQ.............................................
AcFLS                  334  PQ.............................................
VIT_218s0001g03490_Vv  334  PQ.............................................
VIT_218s0001g03470_Vv  334  PQ.............................................
Potra2n4c9667_Pt       365  PQ.............................................
Potra2n4c9684_Pt       365  PQ.............................................
AtFLS1                 334  PLD............................................
AtFLS2                      ...............................................
AtFLS3                 306  LLD............................................
```

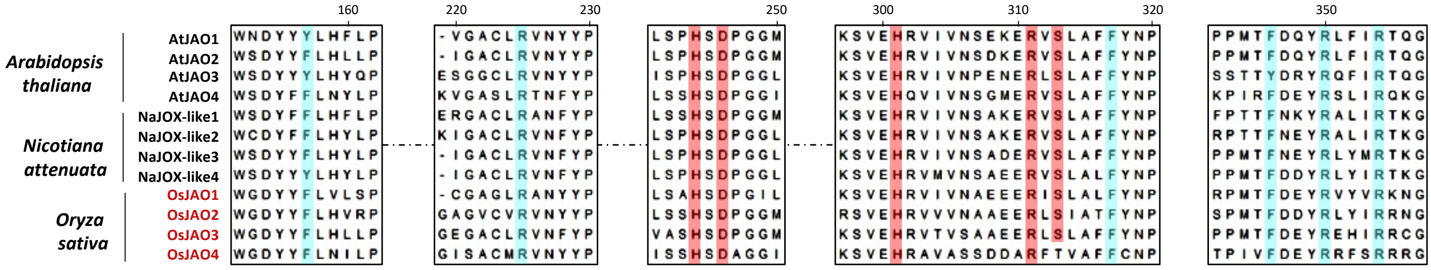

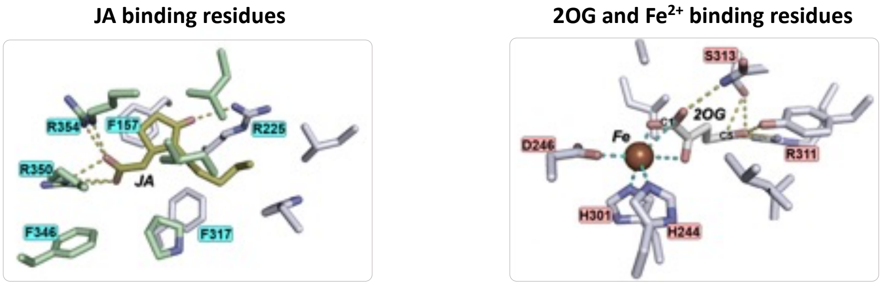

Modified from Zhang et al (2021)
Mol Plant 14, 820-828

Supplementary Figure S2.

Alignment of predicted OsJAO protein sequences with partial sequences of functionally characterized JAO proteins from Arabidopsis and *Nicotiana attenuata*. Conserved amino acid residues identified by Zhang et al (2021) as essential for jasmonic acid (JA) binding are highlighted in blue background; amino acid residues required for binding of co-substrate 2-oxoglutarate (2-OG) and iron (Fe^{2+}) are shown in red. The lower panels show the positions of conserved residues relative to JA substrate (left panel) and 2-oxoglutarate co-substrate (right panel) in the structure of the active site proposed by Zhang et al (2021).

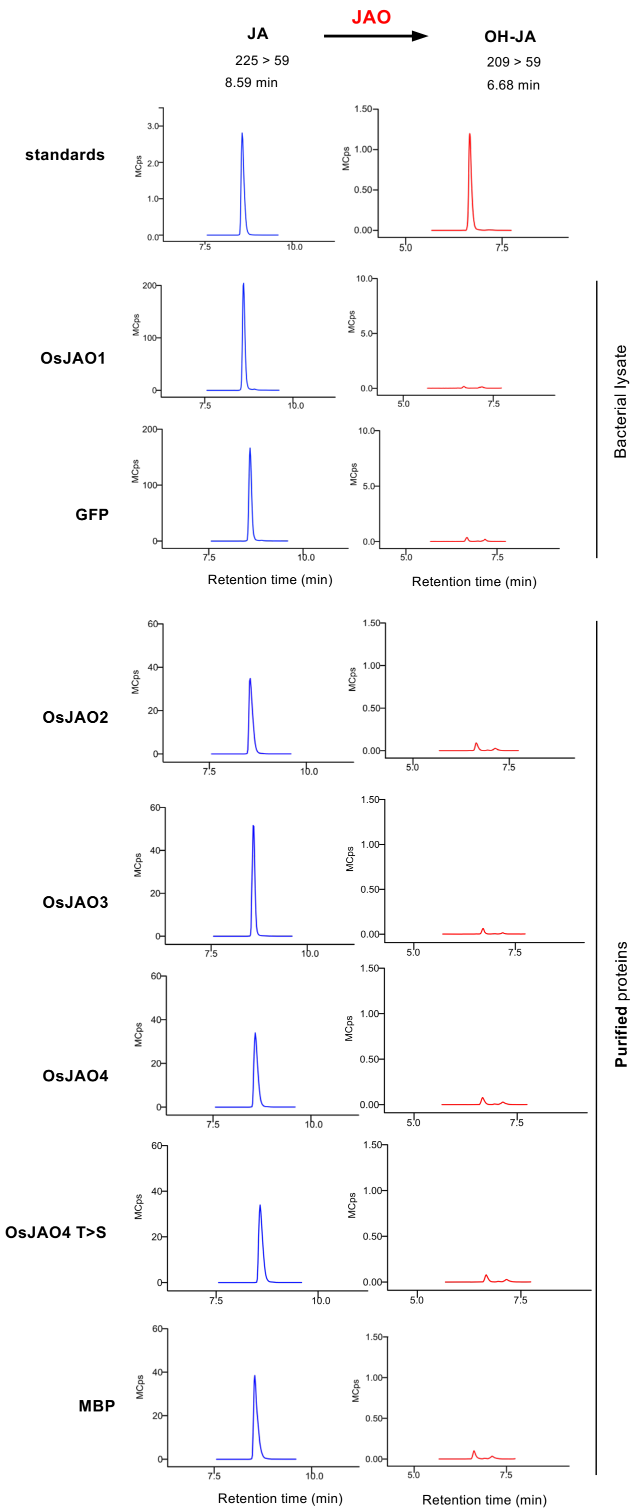

Supplementary Figure S3.

Negative controls for the incubation reactions of recombinant OsJAO from bacterial lysate (OsJAO1 and GFP) or affinity-purified proteins (OsJAO2, 3 and 4) shown in Figure 2a. Reaction mixtures omitted the co-substrate 2-oxoglutarate. Reaction mixtures were analyzed by LC-MS/MS. MCps: megacounts.

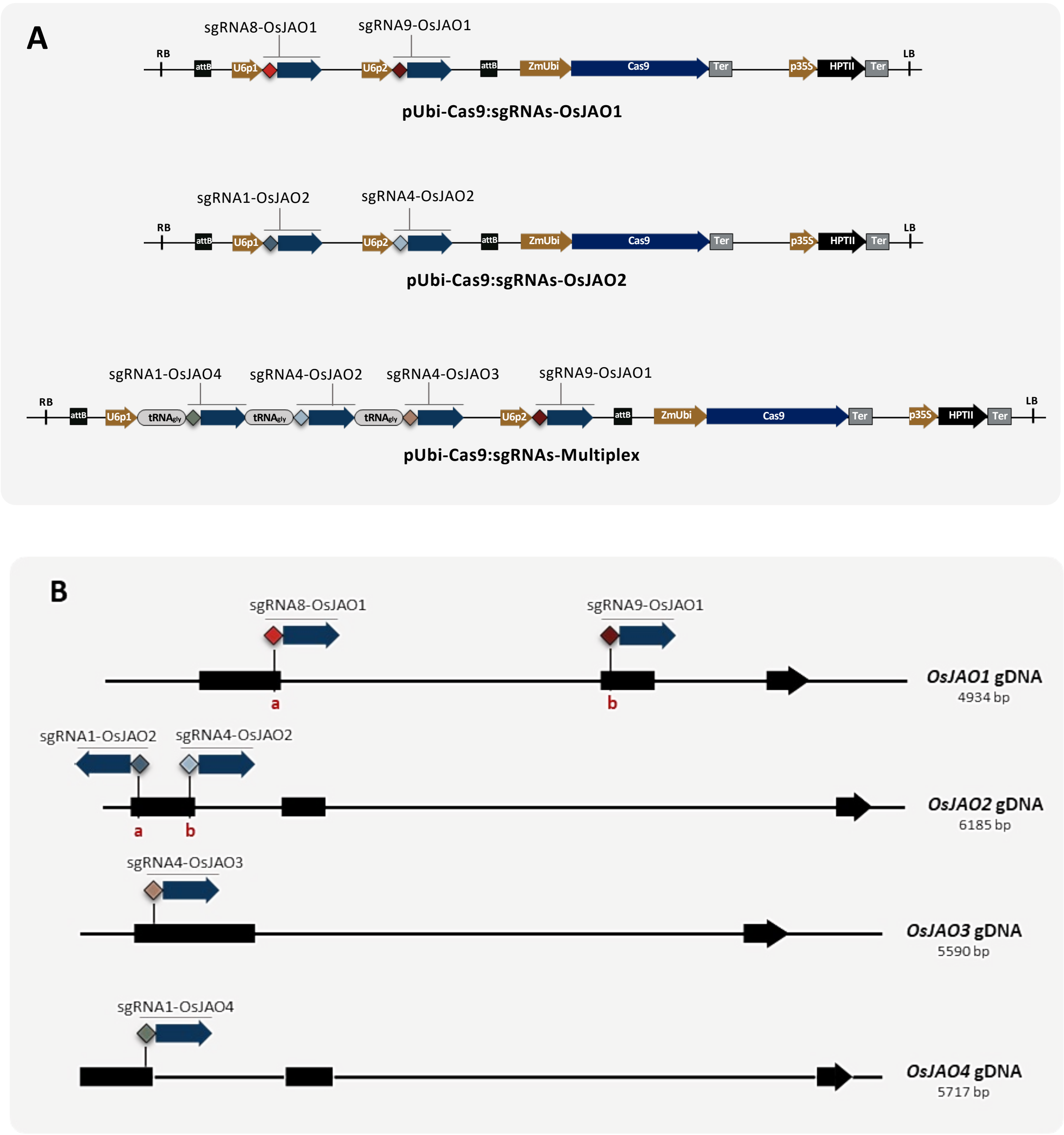

Supplementary Figure S4.
CRISPR-Cas9 plasmid constructs used for generating *Osjao* mutant lines and the positions of target sequences for each guide RNA (sgRNA) in *OsJAO* genes. **A)** Graphical charts of T-DNA regions of the three constructs used to express the mutagenesis CRISPR-Cas9 machinery (Cas9 and gRNA) to produce single *jao1* and *jao2*, and multiple *jao* rice mutant lines. On pUbi-Cas9:sgRNAs-OsJAO1 and pUbi-Cas9:sgRNAs-OsJAO2 constructs, the two sgRNAs selected for the gene were placed under the control of distinct Ubiquitin-6 promoters (U6p1 et U6p2 respectively). On the pUbi-Cas9:sgRNAs-Multiplex construct, the sgRNA-tRNA^{gly} multiplex module was placed under the control of U6p1 for OsJAO2, 3 and 4 and U6p2 for OsJAO1. LB: left border; RB: right border. **B)** Graphical chart of *OsJAO1*, *OsJAO2*, *OsJAO3*, *OsJAO4* gene organization (gDNA), showing the positions of target sites of each sgRNA on the respective genes. Genes are represented by filled rectangles (exons) and solid lines (non-coding sequences). Oriented sgRNAs are depicted by filled arrows and diamonds. a and b designate the positions of target sequences of first and second sgRNA respectively in exons of *OsJAO1* and *OsJAO2* genes.

A

Mutant Plant Lines

gene	indel	knock out	jao2 #27	jao2 #55	jao1.2.3*.4* #67	jao1.2.3*.4 #137	jao1.2.3.4* #11	jao1.2.3.4* #13	jao1.2.3.4 #249	jao1.2.3.4 #170	encoded protein
OsJAO1	-1	yes			■		■	■	■	■	OsJAO1:p.L191Wfs*88
	-2	yes				■					OsJAO1:p.L191Gfs*63
OsJAO2	+1	yes			■		■	■		■	OsJAO2:p.W155Vfs*6
	-1	yes	■			■			■		OsJAO2:p.K155Sfs*19
	-2	yes		■							OsJAO2:p.Y32Tfs*1
OsJAO3	+1	yes						■		■	OsJAO3:p.D62Efs*348
	-1	yes							■		OsJAO3:p.D62Tfs*21
	-3	no			■	■					OsJAO3:p.61PD>H
	-25	yes					■				OsJAO3:p.V55Gfs*20
OsJAO4	+1	yes								■	OsJAO4:p.E158Gfs*5
	-1	yes				■			■		OsJAO4:p.E158Rfs*14
	-3	"potentially" no			■		■	■			OsJAO4:p.E158del

B

2-OG and Fe^{2+} binding residues

Essential JA binding residues

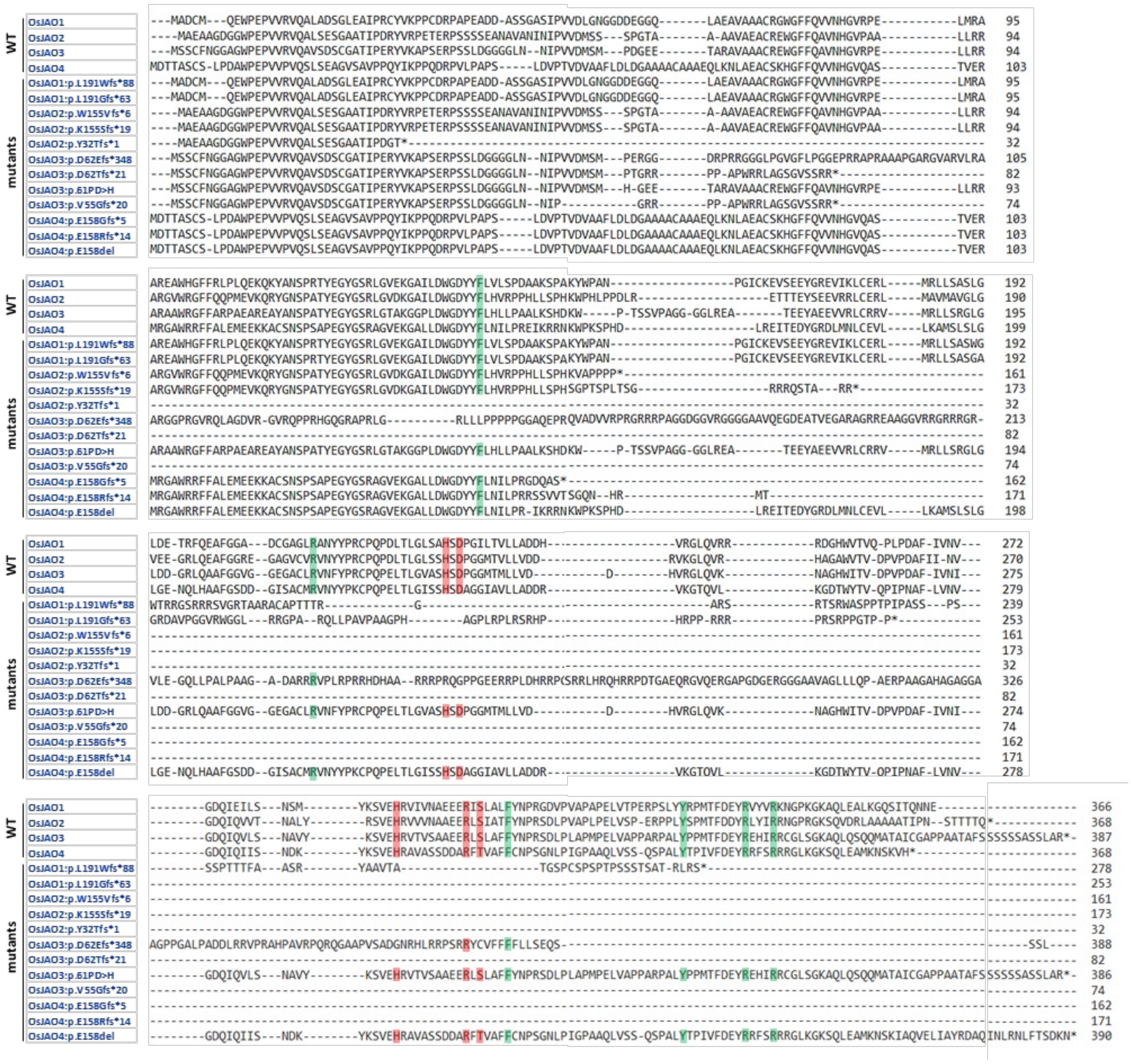

Supplementary Figure S5.

Representation of mutations (indels) identified in *OsJAO* genes in different rice mutant lines in the T1 generation, and the truncated proteins encoded by these mutated genes. **A)** Summary of the nature and combinations of indels in the series of single and multiple *Osjao* mutant lines obtained. The column 'indel' lists the indels detected and the filled rectangles indicate their occurrence in different mutant lines. In black: stabilized indel (homozygote). In columns indicating mutant plant lines, gene alleles marked with an asterisk bear in-frame mutation leading to one amino-acid deletion that may preserve a functional protein. The right column describes the impact of the mutation on the structure of a putative encoded protein, using the nomenclature of Escande and Rouleau (2015). fs: frameshift. del: deletion. In the encoded protein name, the number after the asterisk indicates the number of amino acid residues from the frameshift to the next stop codon. **B)** Alignment of wild-type OsJAO protein sequences with those of their mutant variants potentially expressed in obtained *Osjao* mutant lines. Predicted protein sequences derived from mutated gene sequences were aligned using 'Muscle' software to visualize impact of mutations on protein primary structure. Residues required for JAO activity (2-OG/Fe2+ binding and essential JA binding residues) are depicted with pink and green background respectively and can be checked for presence/absence in mutant proteins.

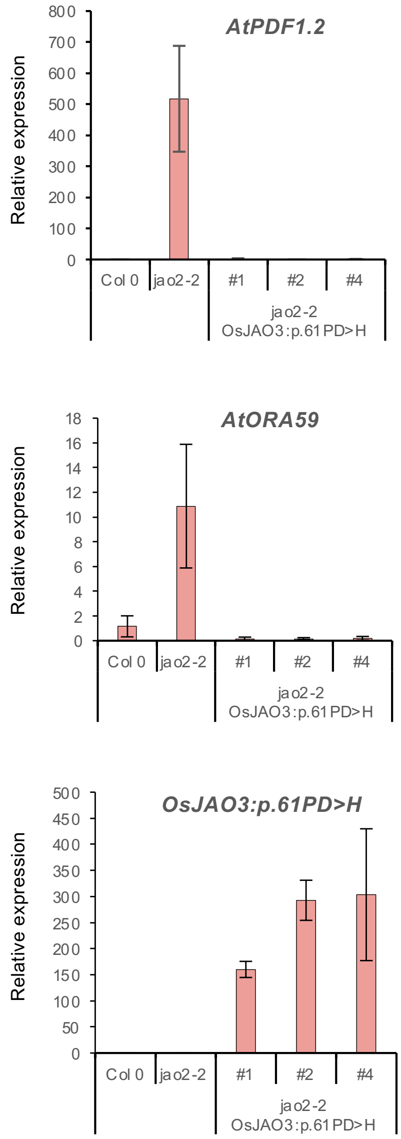

Supplementary Figure S6.
In planta assay of OsJAO3:p.61PD>H functionality. The cDNA encoding OsJAO3:p.61PD>H was cloned from the mutant rice line *jao1.2 #67* and ectopically expressed in Arabidopsis *jao2-2* line under p35S promoter. RNA from three independent T2 transformants along with untransformed WT (Col-0) and *jao2-2* was analyzed for *PDF1.2* marker gene expression. Expression was normalized with signal from *EXP* and *TIP41* housekeeping genes. Histograms show mean ± SEM from three biological replicates.

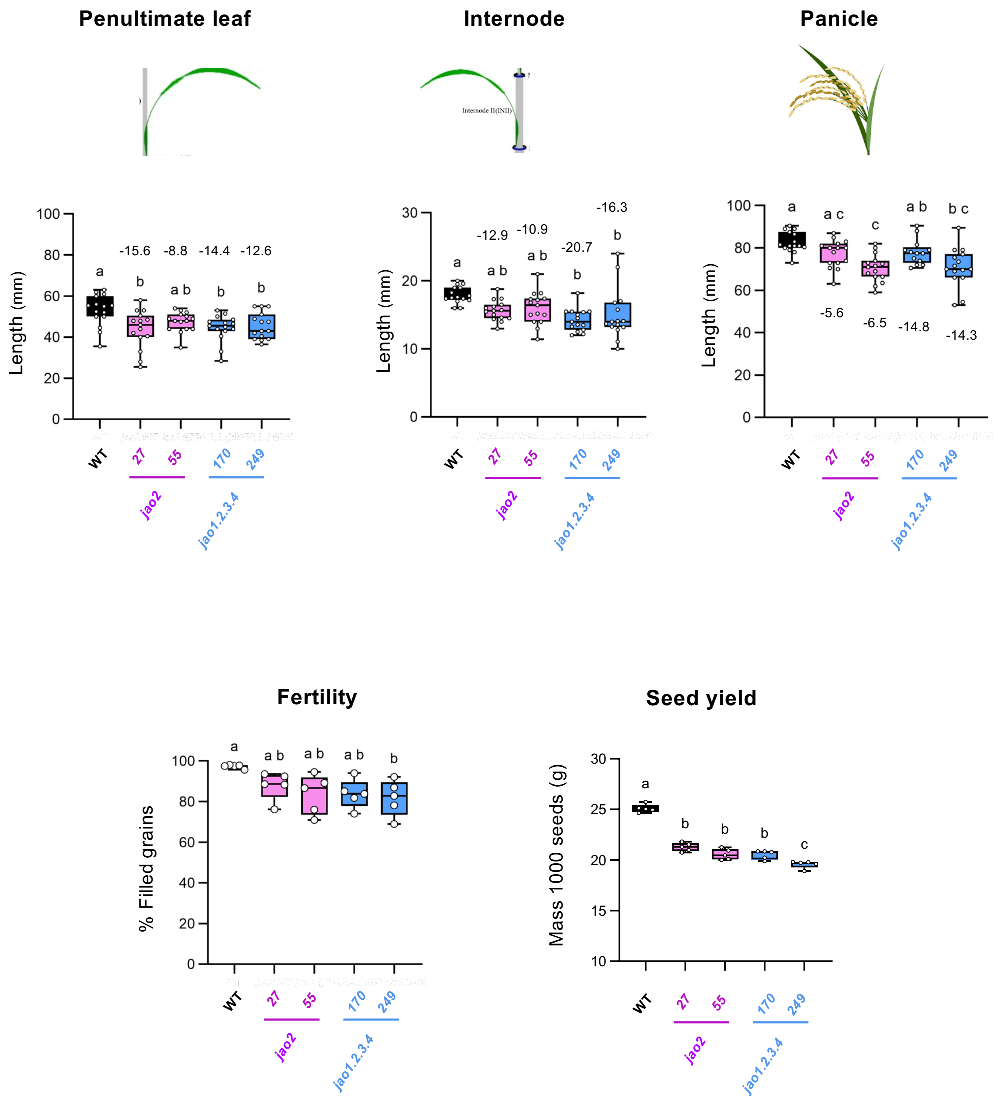

Supplementary Figure S7.

Analysis of the growth parameters of rice mutant lines impaired in *OsJAO* expression at the T3 generation. Plants were grown for 3 months in a greenhouse under a 16h-day/8h-night photoperiod, at 28°C/24°C with a 75% relative humidity. Penultimate leaf, internode and panicle lengths were measured at the mature stage on the 3 highest tillers from five T3 plants for each homozygous osjao line and wild type (WT) plants (n=15). Numbers indicate % mean reduction for each genotype relative to WT. Fertility rate was measured on the same tillers as the ratio between the number of fertile spikelets and the total number of spikelets. Seed yield was measured on the same tillers as the mass of 50 seeds of 5 panicles per genotype. Individual values are depicted by open circles. The different elements in boxes are: center line, median; box limit, upper and lower quartile; wiskers, 1.5x interquartile range; points, outliers. Statistical significance was assessed by one-way ANOVA followed by Tukey post-hoc test with a 95% confidence interval. Different letters above histograms indicate genotypes that are significantly different ($p<0.05$).

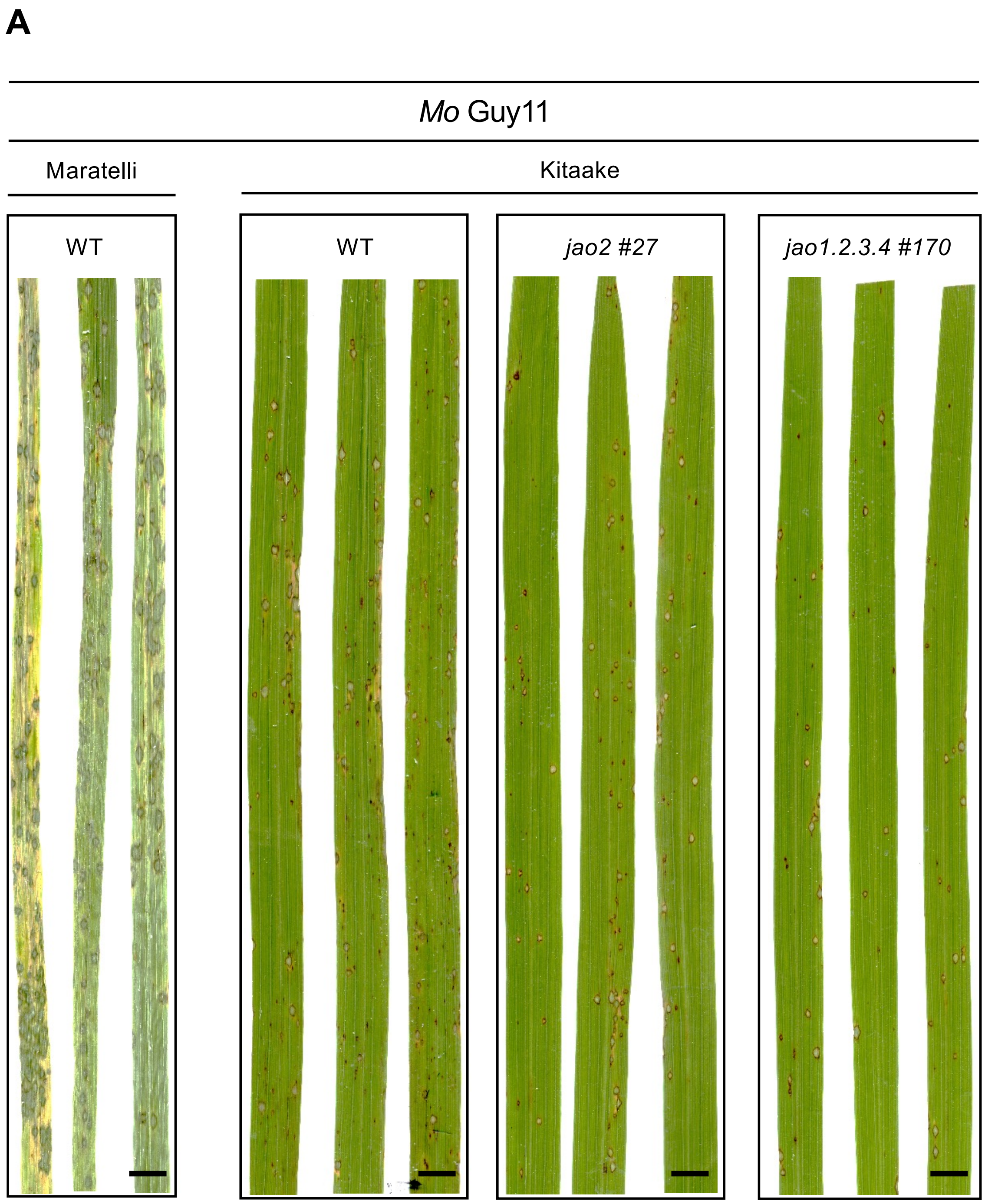

Supplementary Figure S8.

Phenotypic characterization of the interaction of rice *jao* mutant lines with virulent and avirulent strains of the fungal pathogen *Magnaporthe oryzae*.

A) Representative images of leaves from Maratelli WT, Kitaake WT and Kitaake *jao* mutant infection experiment with *Mo* strain Guy11. Images were digitally extracted for comparison. Scale bar = 1 cm. For details see legend of Figure 7.

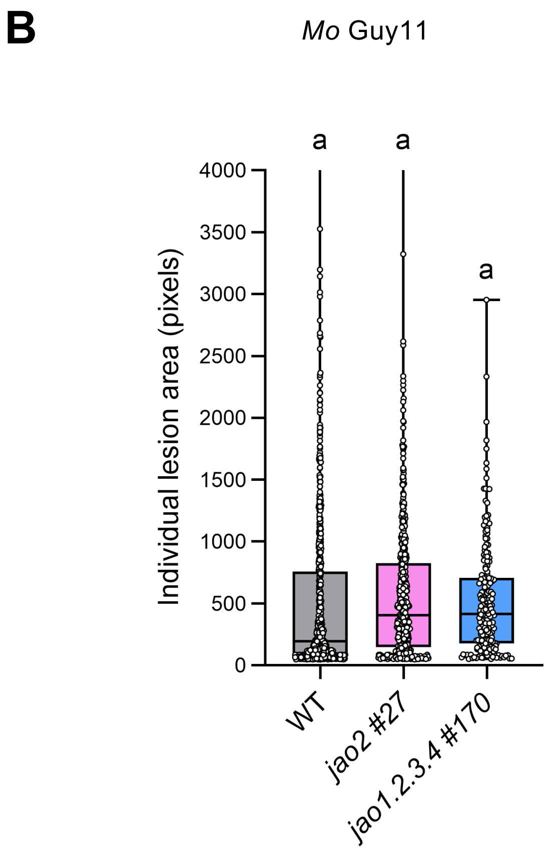

Supplementary Figure S8 (continued)

B) Individual lesion area on leaves of plants inoculated with strains *Mo* Guy11 7 days after inoculation. The boxes represent the second quartile, median, and third quartile. Individual data points are presented as circles. Statistical significance was assessed by two-way ANOVA followed by Tukey post-hoc test with a 95% confidence interval. Different letters above histograms indicate genotypes that are significantly different ($p<0.05$) .

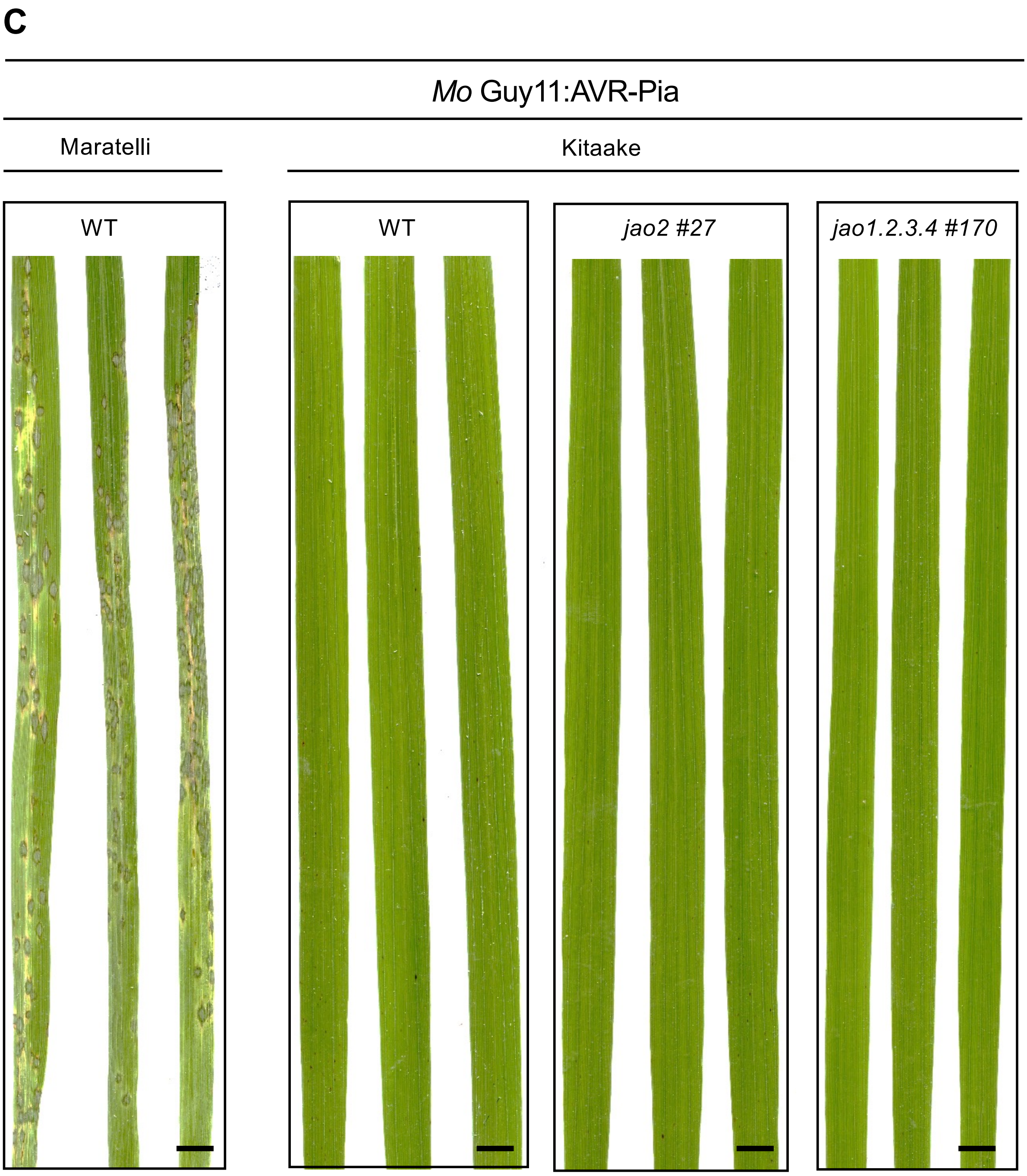

Supplementary Figure S8 (continued)

C) Representative images of leaves from Maratelli WT, Kitaake WT and Kitaake *jao* mutant infection experiment with *Mo* strain Guy11:AVR-Pia. Images were digitally extracted for comparison. Scale bar = 1 cm. For details see legend of Figure 7.

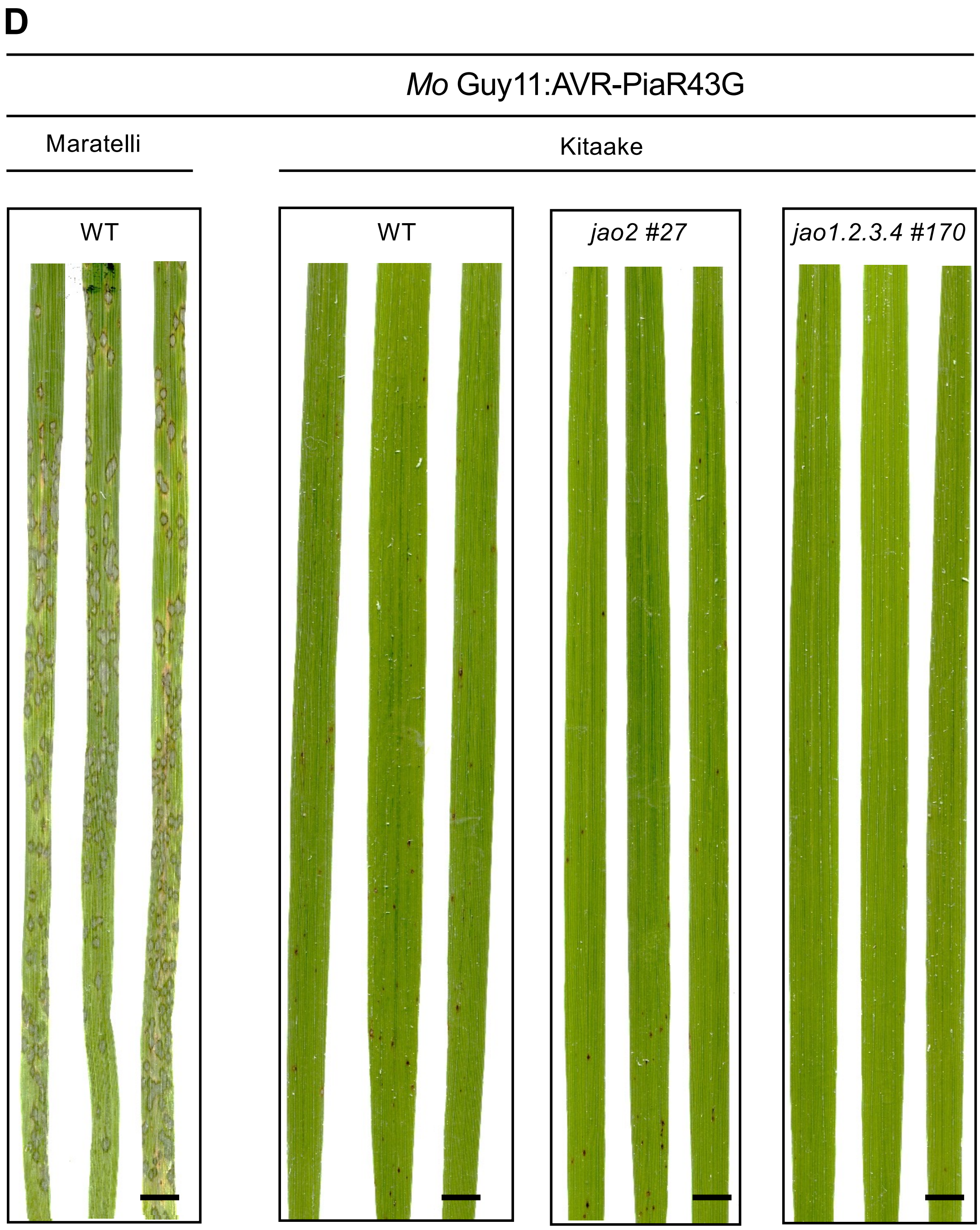

Supplementary Figure S8 (continued)

D) Representative images of leaves from Maratelli WT, Kitaake WT and Kitaake *jao* mutant infection experiment with *Mo* strain Guy11:AVR-PiaR43G. Images were digitally extracted for comparison. Scale bar = 1 cm. For details see legend of Figure 7.

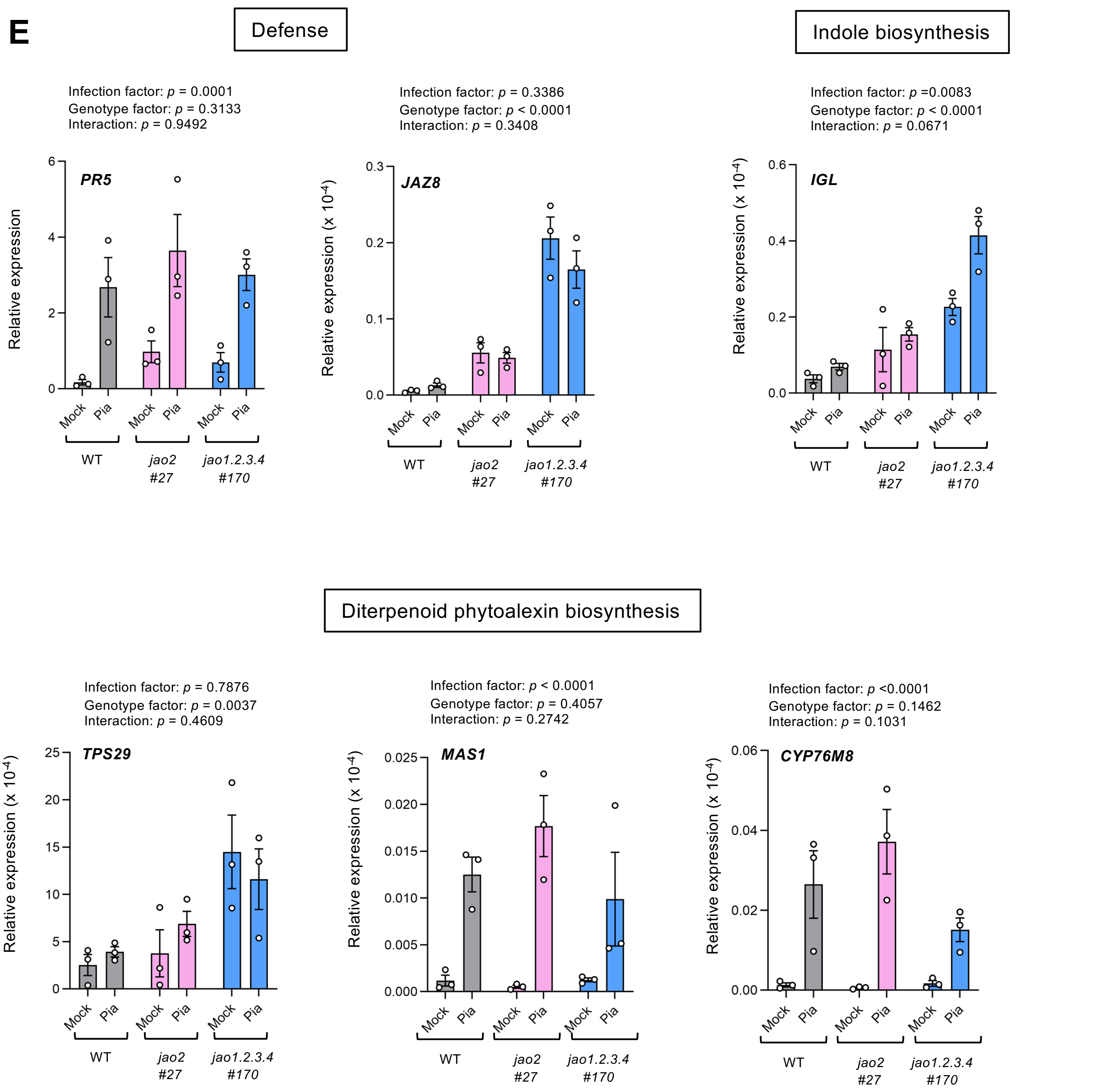

Supplementary Figure S8 (continued)

E) Expression of selected defense-related genes in *jao* mutant plants at 48 h after mock- or Mo Guy11:AVR-PiaR43G- (Pia) inoculation. *PR5*: Pathogenesis-Related 5; *JAZ8*: Jasmonate ZIM domain 8; *IGL*: indole glycerolphosphate lyase; *TPS29*: Terpene synthase 29; *MAS1*: momilactone synthase 1; *CYP76M8*: cytochrome P450 76M8. Expression was normalized with signal from *UBQ5* housekeeping gene. Histograms represent means of 3 biological replicates with SEM. Individual values are depicted by open circles. Statistical significance was assessed by two-way ANOVA. *P*-values of infection, genotype or genotype x infection interaction are indicated.

Supplementary Table S3: Primers used in the study

primer name	primer sequence (5'->3')	gene ID	target
**Assembly of the plasmids pUbi-Cas9 and pENTR4:gRNA4**			
crRNA8_OsJAO1_fw	TGTTGCCAAGAGCCCGGCCAAATAC	*Os01g0832600*	*OsJAO1*
crRNA8_OsJAO1_rv	AAACGTATTTGGCCGGGCTCTTGG		
crRNA9_OsJAO1_fw	GTGTGGGTTGCTGTCAGCGAGCCTG		
crRNA9_OsJAO1_rv	AAACCAGGCTCGCTGACAGCAACC		
crRNA1_OsOsJAO2_fw	TGTTGTCCGGCCTCACGTACCGGTC	*Os05g0127500*	*OsJAO2*
crRNA1_OsOsJAO2_rv	AAACGACCGGTACGTGAGGCCGGA		
crRNA4_OsOsJAO2_fw	GTGTGGGGAGGTGGGGCCACTTGTG		
crRNA4_OsOsJAO2_fw	AAACCACAAGTGGCCCCACCTCCC		
3'_tracrRNA-tRNA_fw	GCACCGACTCGGTGCAACAAAGCACCAGTGGTCTAGTGGTAGAA TAGTACCCTG	-	tracrRNA-tRNA^{gly} fragment
tRNA_rv	CTGCCATGCACCAGCCGGGAATCGAACCCGGGTCTGTACCGTG GCAGGGTACTATTCTAC		
tracrRNA_fw	GGAGACCGAGGTCTCGGTTTTAGAGCTAGAAATA		
tracrRNA_rv	GCACCGACTCGGTGCCAC		
BsaI-tRNA1_fw	GAGGTCTCTTGTTAACAAAGCACCAGTG	-	Polycistronic tRNA-sgRNA sequence
BsaI-crRNA1_OsJAO4-tRNA2_rv	TAGGTCTCCCAAGCGTCGTAATGCACCAGCCGGGAA		
BsaI-crRNA1_OsJAO4-tracrRNA_fw	TAGGTCTCCCTTGATCTCCCTGTTTTAGAGCTAGAA		
BsaI-crRNA4_OsJAO2-tRNA3_rv	CGGGTCTCCGCCCCACCTCCCTGCACCAGCCGGGAA		
BsaI-crRNA4_OsJAO2-tracrRNA_fw	TAGGTCTCCGGGCCACTTGTGGTTTTAGAGCTAGAA		
BsaI-crRNA4_OsJAO2-tRNA_rv	CGGGTCTCCAAACCGTCCGGCATTGACATGTCCTGCACCAGCC GGGAA		
**Assembly of the plasmids pHGWA, pEAQ and pETGG**			
sn77-OsJAO1_SapI-fw	TAAGCTCTTCGGGCATGGCGGACTGCATG	*Os01g0832600*	*OsJAO1*
sn78-OsJAO1_SapI-rv	TAAGCTCTTCGTCATTATTCGTTGTTTTGGGTGATTGA		
sn21-OsJAO2_attB1-fw	GGGGACAAGTTTGTACAAAAAAGCAGGCTATATGGCGGAGGCGG	*Os05g0127500*	*OsJAO2*
sn22-OsJAO2_attB2-rv	GGGGACCACTTTGTACAAGAAAGCTGGGTGTTATTGAGTGGTGG TAGTAGAATTAGGTAT		
sn81-OsJAO2_SapI-fw	TAAGCTCTTCGGGCATGGCGGAGGC		
sn82-OsJAO2_SapI-rv	TAAGCTCTTCGTCATTATTGAGTGGTGGTAGTAGAA		
sn79-OsJAO3_SapI-fw	TAAGCTCTTCGGGCATGAGCAGCTGCTTCA	*Os03g0289800*	*OsJAO3*
sn80-OsJAO3_SapI-rv	TAAGCTCTTCGTCACTAGCGAGCTAGACTGCTC		
sn70-OsJAO4_SapI-fw	TAAGCTCTTCGGGCATGGACACCACGGCAA	*Os11g0437800*	*OsJAO4*
sn72-OsJAO4_SapI-rv	TAAGCTCTTCGTCATCAGTGCACCTTGCTGTTC		
sn170-OsJAO4S_SapI-fw	TAAGCTCTTCGGGCATGGACACCACAGCAT	-	*OsJAO4S*
sn171-OsJAO4S_SapI-rv	TAAGCTCTTCGTCATCAGTGCACCTTACTG		
**Genotyping of *jao* mutants**			
pUbi-Cas9_rv	ACGACAATCTGATCCAAGCT	-	T-DNA_pUbi-Cas9
pUbi-Cas9_fw	GTCACGCTGCACTGCAGGCA		
sn195-OsJAO1_HRM-fw	GAGTACGGGAGAGAGGTGAT	*Os01g0832600*	*OsJAO1*
sn196-OsJAO1_HRM-rv	CGAACGCCTCCTGGAAC		
sn180-OsJAO1_Seq-fw	GGAGAAGCAGAAGTACGCCAA		
sn181-OsJAO1_Seq-rv	CATGCACATGGGCAAAGCTAAA		
sn182-OsJAO1_Seq-fw	TGCCATATTAGGTTTAGCGCATTA		
sn183-OsJAO1_Seq-rv	CACCGCGGGTAGTAGTTGG		
sn176-OsJO2_Seq-fw	AGTGGCTAGCTGAGTGATCG	*Os05g0127500*	*OsJAO2*
sn177-OsJAO2_Seq-rv	GTGGATGAGTGATGTGTACCTGA		
sn193-OsJAO3_HRM-fw	GGCCTGAACAATATCCCGGT	*Os03g0289800*	*OsJAO3*
sn194-OsJAO3_HRM-fw	TTCACCGCCTGGAAGAAACC		
sn178-OsJAO3_Seq-fw	GCGAGTGCATGACGTGCTA		
sn207-OsJAO3_Seq-rv	TGCATATACAAATTGCGGCGG		
sn191-OsJAO4_HRM-fw	TCTCCTTGACTGGGGTGACT	*Os11g0437800*	*OsJAO4*
sn192-OsJAO4_HRM-fw	TGGCCACTTGTTACGACGCT		
sn174-OsJAO4_Seq-fw	TGCCCTAGAGATGGAGGAGA		
sn175-OsJAO4_Seq-rv	ATCTCGCCAAATTAAGTTGAGGTTT		
FW_amplif pENTR4 constrct XhoI	TCTAGATATCTCGAGCAGAATTGCCCTTCGAAGGGA	-	-
RV_amplif pENTR4 constrct BamHI	CAGTCGACTGGATCCCTTAAGAACGAACTAAGCCGG	-	-
**RT-qPCR**			
JAZ9_fw	TTGATGACTTCCCAGCTGAGAA	*Os03g0180800*	*OsJAZ9*
JAZ9_rv	GCGCTGTGGAGGAACTCTTG		
OsNOMT_fw	CTACCTACATCTTCACCAACGT	*Os12g0240900*	*OsNOMT*
OsNOMT_rw	GAGACTGAGAAGAGGAAACGAA		
OsTPS30_fw	GGGCTCGAGTGAAGTACCAG	*Os08g0167800*	*OsTPS30*
OsTPS30_rv	AGGTCGTTTATCTCAGTCTCGT		
RBBI2-2_fw	GAGATGGTGGACTCGTGGTC	*Os01g0123900*	*OsRBBI2-2*
RBBI2-2_rv	AGTTGTGCATTTCAGGTGTGC		
UBQ5_fw	ACCACTTCGACCGCCACTACT	*Os01g0328400*	*OsUBQ5*
UBQ5_rv	ACGCCTAAGCCTGCTGGTT		
UBQ10_fw	GAGCCTCTGTTCGTCAAGTA	*Os02g0161900*	*OsUBQ10*
UBQ10_rv	ACTCGATGGTCCATTAAACC		
sn5-OsJAO1_qPCR-fw	GTCGGCGACCAGATTGAG	*Os01g0832600*	*OsJAO1*
sn6-OsJAO1_qPCR-rv	AGAGTGCGAGCGAGATGC		
sn3-OsJAO2_qPCR-fw	GGCGACCAAATTCAGGTG	*Os05g0127500*	*OsJAO2*
sn4-OsJAO2_qPCR-rv	GAAGGTCGCGATCGACAG		
sn382-OsJAO3_qPCR-fw	ATCACCGTCGACCCTGTTC	*Os03g0289800*	*OsJAO3*
sn383-OsJAO3_qPCR-rv	CGTTGCTCAGCACCTGTATCT		
sn231-OsJAO4_qPCR-fw	GGAGATCAAGCGTCGTAACA	*Os11g0437800*	*OsJAO4*
sn232-OsJAO4_qPCR-rv	AGCGACATGGCTTTGAGTAG		
PDF1.2_fw	CACCCTTATCTTCGCTGCTCTT	*AT5G44420*	*AtPDF1-2*
PDF1.2_rv	TACACTTGTGTGCTGGGAAGAC		
EXP_qPCR-fw	GAGCTGAAGTGGCTTCAATGAC	*At4g26410*	*AtEXP*
EXP_qPCR-rv	GGTCCGACATACCCATGATCC		
TIP41_qPCR-fw	GTGAAAACTGTTGGAGAGAAGCAA	*At4g34270*	*AtTIP41*
TIP41_qPCR-rv	TCAACTGGATACCCTTTCGCA		

Supplementary Table S3 (continued)

Gene	crRNA	Sequence
OsJAO1	crRNA8_OsJAO1	CCAAGAGCCCGGCCAAATAC
OsJAO1	crRNA9_OsJAO1	GGTTGCTGTCAGCGAGCCTG
OsJAO2	crRNA1_OsJAO2	TCCGGCCTCACGTACCGGTC
OsJAO2	crRNA4_OsJAO2	GGGAGGTGGGGCCACTTGTG
OsJAO3	crRNA4_OsJAO3	GGACATGTCAATGCCGGACG
OsJAO4	crRNA1_OsJAO4	TTACGACGCTTGATCTCCCTCGG
